# Assessment of temperature optimum signatures of corals at both latitudinal extremes of the Red Sea

**DOI:** 10.1093/conphys/coac002

**Published:** 2022-02-18

**Authors:** Guilhem Banc-Prandi, Nicolas R Evensen, Daniel J Barshis, Gabriela Perna, Youssouf Moussa Omar, Maoz Fine

**Affiliations:** The Goodman Faculty of Life Sciences, Bar-Ilan University, Ramat-Gan 52900, Israel; The Interuniversity Institute for Marine Sciences, Eilat, 88103, Israel; Department of Biological Sciences, Old Dominion University, Norfolk, VA, USA; Department of Biological Sciences, Old Dominion University, Norfolk, VA, USA; Department of Biology, University of Konstanz, Konstanz, Germany; Center for Studies and Scientific Research of Djibouti, Route de l’Aéroport, BP 1000, Djibouti; The Goodman Faculty of Life Sciences, Bar-Ilan University, Ramat-Gan 52900, Israel; The Interuniversity Institute for Marine Sciences, Eilat, 88103, Israel

**Keywords:** thermal adaptation, Red Sea, Gulf of Tadjoura, Gulf of Aqaba, coral reefs, Coral bleaching

## Abstract

Rising ocean temperatures are pushing reef-building corals beyond their temperature optima (*T_opt_*), resulting in reduced physiological performances and increased risk of bleaching. Identifying refugia with thermally resistant corals and understanding their thermal adaptation strategy is therefore urgent to guide conservation actions. The Gulf of Aqaba (GoA, northern Red Sea) is considered a climate refuge, hosting corals that may originate from populations selected for thermal resistance in the warmer waters of the Gulf of Tadjoura (GoT, entrance to the Red Sea and 2000 km south of the GoA). To better understand the thermal adaptation strategy of GoA corals, we compared the temperature optima (*T_opt_*) of six common reef-building coral species from the GoA and the GoT by measuring oxygen production and consumption rates as well as photophysiological performance (i.e. chlorophyll fluorescence) in response to a short heat stress. Most species displayed similar *T_opt_* between the two locations, highlighting an exceptional continuity in their respective physiological performances across such a large latitudinal range, supporting the GoA refuge theory. *Stylophora pistillata* showed a significantly lower *T_opt_* in the GoA, which may suggest an ongoing population-level selection (i.e. adaptation) to the cooler waters of the GoA and subsequent loss of thermal resistance. Interestingly, all *T_opt_* were significantly above the local maximum monthly mean seawater temperatures in the GoA (27.1°C) and close or below in the GoT (30.9°C), indicating that GoA corals, unlike those in the GoT, may survive ocean warming in the next few decades. Finally, *Acropora muricata* and *Porites lobata* displayed higher photophysiological performance than most species, which may translate to dominance in local reef communities under future thermal scenarios. Overall, this study is the first to compare the *T_opt_* of common reef-building coral species over such a latitudinal range and provides insights into their thermal adaptation in the Red Sea.

## Teaser

Thermal performance assays were conducted on six common reef-building coral species from the northern and the southern Red Sea (2000 km apart). Most species displayed similar temperature optima (*T_opt_)*, suggesting an exceptional continuity in their physiological performances across such a large latitudinal range.

## Introduction

Temperature is one of the main factors shaping the biology and ecology of organisms across all ecosystems (Angilletta, 2009b). In the context of rapid anthropogenic climate change, temperature variability constitutes the main threat to a wide range of habitats (e.g. Doney *et al*., 2012; Pontavice *et al*., 2020; Malhi *et al*., 2020). Among others, coral reefs are known for their high sensitivity to such stress (Hoegh-Guldberg, 1999) and have suffered a drastic decline in the recent decades (Hughes *et al*., 2017; Hughes *et al*., 2018; Lough *et al*., 2018). Assessing the responses of corals to temperature variability can assist in identifying mechanisms involved in local thermal adaptation or acclimatization, and therefore may allow to predict ‘winners’ and ‘losers’ under future conditions (e.g. Loya *et al*., 2001; Rohr *et al*., 2018; van Woesik *et al*., 2011). Thermal acclimatization of corals can occur when prolonged exposure to elevated temperature leads to an increase of critical thermal maximum (mean upper limit of performance) or temperature optima (*T_opt_*) of a biological trait within their life span (Sinclair *et al*., 2016). In this context, *T_opt_* refers to the temperature at which a specific physiological trait is maximum (e.g. photosynthesis), while thermal threshold refers to a temperature limit, above which such trait may start crashing (Padfield *et al*., 2021). Just like thermal thresholds, above which corals are predicted to undergo bleaching, *T_opt_* can vary between species within regions (e.g. Gould *et al*., 2021; Jurriaans and Hoogenboom, 2019) and across regions for similar species (e.g. Sawall *et al*., 2014; Ulstrup *et al*., 2006). Moreover, *T_opt_* of corals also depends on various factors, such as the genotype of the coral host (e.g. Dilworth *et al*., 2021; Dixon *et al*., 2015) or its symbionts (e.g. Berkelmans and van Oppen, 2006; Ulstrup *et al*., 2006; Jones *et al*., 2008), the density and performances of the symbionts (Madin *et al*., 2016) and the holobiont (both host and symbionts) acclimatization history (Ainsworth *et al*., 2016; Palumbi *et al*., 2014).

The coral thermal breadth of performance, the range of temperatures over which a coral performs optimally for a given biological trait, may limit its acclimatization or adaptation capabilities in a warming environment (Raymond and Kingsolver, 1993; Angiletta, 2009a). Thermal performance curves (TPCs) quantify how a biological trait such as growth, photosynthesis and respiration rates varies with temperature and are commonly used to assess thermal acclimatization and adaptation. With extensive evidence of organismal acclimatization or adaptation across spatial temperature gradients, ranging from local to regional scales (e.g. Castillo *et al*., 2012; Oliver and Palumbi, 2011), TPCs are used to assess the range of survivable temperatures of an organism and characterize its response to temperature variability within this range (Padfield *et al*., 2021; Sinclair *et al*., 2016). As such, TPCs can assist in predicting the evolution in species richness and diversity and the functional impacts of elevation of seawater temperatures (Aichelman *et al*., 2019; Gould *et al*., 2021; Padfield *et al*., 2021; Silbiger *et al*., 2019). Recent studies implementing the TPC approach succeeded in quantifying differences in temperature acclimatization of various coral species between environments with different temperature regimes (e.g. Aichelman *et al*., 2019; Gould *et al*., 2021; Jurriaans and Hoogenboom, 2019; Silbiger *et al*., 2019; Rodolfo-Metalpa *et al*., 2014). For example, the Caribbean reef-building coral *Orbicella franski* displayed higher metrics derived from TPCs [*T_opt_*, activation energy *Eh*, rate at a standardized temperature *b(T_c_)*] in the warmer waters of Panama compared to populations of the same species acclimatized to the cooler waters of Bermuda (Silbiger *et al*., 2019). Similarly, *Astrangia poculata* was shown to respond differently to temperature variability across symbiotic states and latitudes, reflecting distinct evolutionary strategies of this species along the East Coast of the USA (Aichelman *et al*., 2019).

Despite being one of the world’s warmest and most saline seas (up to 34°C and 41 psu; Edwards and Head, 1986), the Red Sea hosts some of the richest and most diverse coral reef ecosystems (Dibattista *et al*., 2016), with high similarity among coral assemblages along its latitudinal gradient (Riegl *et al*., 2012). Extending over 2270 km from 30°N in the Gulf of Suez to 12°N in the strait of Bab el Mandab, the Red Sea displays strong north–south gradients of temperature (north: 20–27°C; south: 28–34°C; winter–summer), salinity (37–41 psu) and primary productivity (0.5–4.0 mg m^−3^ chlorophyll *a*) (Raitsos *et al*., 2013; Sawall and Al-sofyani, 2015). The central and southern Red Sea have experienced sporadic bleaching events (e.g. 1998, 2010 and 2015 in Saudi Arabia; Monroe *et al*., 2018; Decarlo, 2020), with summer sea surface temperatures (SSTs) reaching up to 33–34°C (Sawall *et al*., 2014). Yet, bleaching has not been observed in the northern Red Sea and Gulf of Aqaba (GoA), despite a 0.4–0.5°C increase in summer SSTs per decade over the past 30 years (Osman *et al*., 2018) and multiple thermal anomalies.

Corals from the GoA display high thermal resistance [high thermal threshold relative to their local maximum monthly mean (MMM)] in response to experimental heat stress (Bellworthy and Fine, 2017; Evensen *et al*., 2021; Fine *et al*., 2013; Savary *et al*., 2021; Voolstra *et al*., 2021) and increased primary productivity when exposed to 11 degree heating weeks (DHWs) (Krueger *et al*., 2017), conditions that would typically incur severe bleaching and mortality (Hughes *et al*., 2018). This suggests that GoA corals live much below their upper bleaching threshold as opposed to corals in the central and southern Red Sea (Fine *et al*., 2013; Osman *et al*., 2018). Such high thermal thresholds are hypothesized to be linked to historical selection for heat resistance during successive re-colonization events through a thermal bottleneck at the Bab el Mandab strait (southern Red Sea) following the last glacial maximum (Fine *et al*., 2013). The few studies comparing the responses of corals to heat stress along a latitudinal gradient in the Red Sea (Sawall *et al*., 2014; Grottoli *et al*., 2017; Osman *et al*., 2018; Voolstra *et al*., 2021; Evensen *et al*, unpublished) all indicate increasing thermal thresholds from north to south with increasing MMM SSTs. Yet, no quantification of *T_opt_* has ever been reported for Red Sea corals, which constitutes a significant knowledge gap when aiming at understanding thermal adaptation or acclimatization strategies of corals across the Red Sea latitudinal gradient.

**Figure 1:**
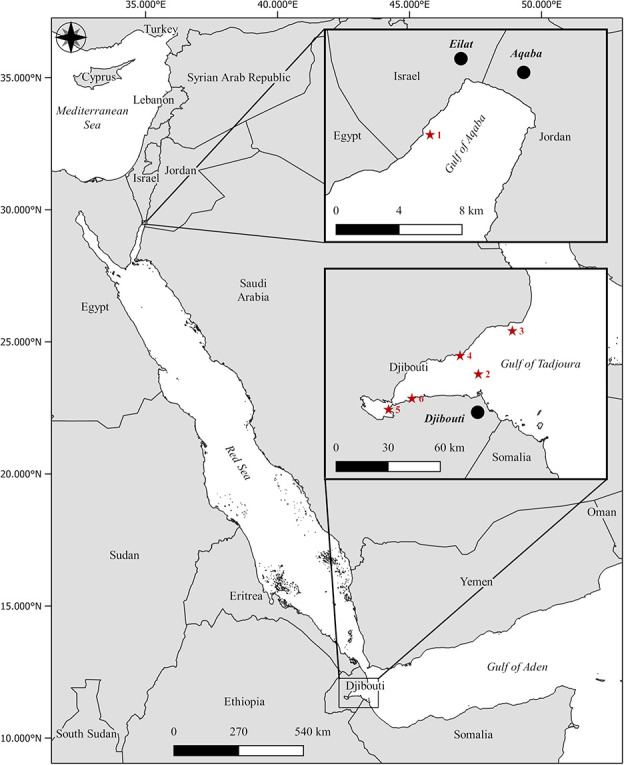
Map of the Red Sea region, highlighting the different sampling sites. In the GoA, the coral nursery of the IUI for Marine Sciences of Eilat, Israel, in the northern Red Sea (1), and five sites (2–6) in the GoT at the southern entrance of the Red Sea. Cities, bold italic; seas, italic. Red stars refer to the exact sampling sites (GoA, 1; GoT, 2–6). GPS coordinates are available in the supplementary materials (Supplementary Table S1).

The Gulf of Tadjoura (GoT, Djibouti) is located 70 km south of the Bab el Mandab strait and 2000 km south of the GoA (Fig. 1). This semi-enclosed sea is subject to Red Sea influence in the North and Indian Ocean in the East (Youssouf *et al*., 2016). Located at the junction between the Red Sea and the Gulf of Aden, it hosts a number of endemic species from these two large biogeographical regions (56 coral genera; Youssouf *et al*., 2016; Cowburn *et al*., 2019) and is hypothesized to constitute the original source of thermally resistant coral populations, selected for their resistance to the elevated temperatures of the southern Red Sea and currently found in the GoA (Fine *et al*., 2013). Summer MMM SST in the GoT is ~30.9°C (1982–2016; Cowburn *et al*., 2019), which would indicate a predicted bleaching threshold of 31.9°C (MMM + 1°C; sensu Coral Reef Watch), compared to the GoA, with an MMM of 27.1°C (2008–2018; Israel National Monitoring Program) and an experimentally assessed bleaching threshold of ~33°C (MMM + 6°C; Krueger *et al*., 2017). Cowburn *et al*. (2019) reported that the latest major coral bleaching event documented in the GoT occurred in 1998, when cumulative thermal stress exceeded 8 DHWs (Liu *et al*., 2006). Little is known about the physiological characteristics of common GoT corals, with regards to what is currently established for similar species in the GoA (e.g. photophysiology, symbiont cell density). Despite dissimilar environmental conditions, the two locations host healthy coral communities that share a number of coral species (Cowburn *et al*., 2019; Fine *et al*., 2013), providing an opportunity to experimentally contrast the physiological performances and thermal stress responses of corals at both ends of the Red Sea’s latitudinal gradient. Here, we compare the *T_opt_* (based on dark respiration and gross photosynthesis rates) of six common reef-building coral species between the GoA and the GoT and describe their photophysiological performances in response to a short heat stress to better understand the thermal adaptation strategy of GoA corals.

## Materials and methods

### Study locations

The study was performed in March 2020 in the GoT, Djibouti (N 11.71444 – E 43.01226; Fig. 1; Supplementary Table S1) ~70 km south of the Bab el Mandab strait, on board the M/V Deli and replicated a week later in Eilat, Israel, at the northern tip of the GoA (N 29.50232 – E 34.91703; Fig. 1; Supplementary Table S1). In the GoT, each coral species was collected from a different reef site due to logistical constraints (Fig. 1; Supplementary Table S1), whereas in the GoA, all species were collected from a single site on the Israeli coast, at the Interuniversity Institute (IUI) for Marine Sciences (Fig. 1; Supplementary Table S1). The average temperature in the GoT during the 1-week experiment was 28± 0.5°C and 22± 0.5°C in the GoA.

### Experimental design

Eight 4-cm-long coral fragments were collected at 5–8 m depth from eight distinct scleractinian colonies (one fragment per colony) of five Anthozoan coral species, *Stylophora pistillata*, *Acropora muricata*, *Porites lobata* (3-cm diameter cores), *Seriatopora hystrix* and *Pocillopora verrucosa*, and from the Hydrozoan coral *Millepora dichotoma*. Samples were transferred into temperature-controlled tanks for 30 minutes to recover from handling stress, at the respective temperature of the sampling site (referred to as ‘ambient’ treatment), followed by initial measurements of photophysiological performances (chlorophyll fluorescence, see below). Fragments were then individually placed in metabolic chambers and TPCs based on dark respiration (*R_dark_*) and gross photosynthetic (*Pg*) rates (see below) were performed, consisting of a series of successive 20-minute incubations at increasing temperatures (28, 30, 32 and 34°C in the GoT; 22, 24, 26, 28, 30, 32 and 34°C in the GoA), with temperature ramping rates of 2°C/10 minutes between holds. At each temperature, measurements of *R_dark_* were conducted first for 10 minutes or until the rates of oxygen concentration evolution had been constant for at least 5 minutes, followed by net photosynthesis (*Pn*) with a similar approach. Once at 34°C, the photophysiological performances were measured again (referred to as the ‘elevated temperature’ treatment). Fragments were then processed for symbiont cell density and surface area measurements.

### Pulse amplitude modulated fluorometry

The quantum yield of photosystem II (PSII) of the fragments from each species in both locations were measured using the Maxi version of the Imaging-PAM (WALZ GmbH, Effeltrich, Germany) to estimate their photophysiological performances in response to a short heat stress. Following a 15-minute dark acclimation period, rapid light curves (RLCs) were generated to assess the sensitivity of PSII to changing photosynthetically active radiation (PAR) consisting of sequences of thirteen 20-second intervals of increasing light intensities ranging from 0 to 701 μmol quanta m^−2^ s^−1^, with each interval followed by a saturating pulse (Supplementary Figs S1–S4). The effective photosynthetic efficiency (YII) and the non-photochemical quenching (NPQ) were derived using the Imaging PAM software (ImagingWin v2.41a). For the values of NPQ to fall in the range of the false colour scale of the display system (0 to 1), NPQ was divided by 4 by the software and referred to as NPQ/4 for the downstream analysis. Additionally, the maximum photochemical efficiency (*F_v_/F_m_*) was calculated as (*F_m_* − *F_0_*/*F_m_*), with *F_m_* and *F_0_* corresponding to the maximum and minimum fluorescence emitted by the coral endosymbiont after dark acclimation, respectively. The maximum NPQ/4 (*NPQ/4_max_*) was obtained from the RLCs by selecting the values of NQP/4 at maximum PAR = 701 μmol quanta m^−2^ s^−1^. The relative electron transport rate (*rETR*) was obtained as (YII)*PAR*0.5 (Ralph and Gademann, 2005). The maximum rETR (*rETR_max_*, the maximum yield for each sample), the relative initial photosynthetic rate (*alpha*, the slope of the curve in the light-limiting region, indicative of the ability of PSII to maximize yield before the onset of saturation; Ralph and Gademann, 2005) and the compensation point (*iK* = *rETR_max_/alpha*, the minimum saturating irradiance, above which NPQ dominates over fluorescence quenching; Ralph and Gademann, 2005) were extracted from the *rETR* RLCs using the ‘Phytotools’ package from the statistical software R (version 3.6.2). The function ‘fitPGH’ was used to calculate photosynthetic-irradiance (PE) parameters (*alpha*, *beta*, *ps*) and fit statistics for PE or RLC data using the model of Platt *et al*. (1980). When the photosynthetic endosymbionts of the coral experience stress, changes in quantum pathways and a decrease in efficiency of the photosystems may occur (Hill *et al*., 2004). Thus, decreases in *F_v_/F_m_*, *rETR_max_*, *NPQ/4_max_*, *alpha* and *iK* indicate malfunctions in PSII, which may result in a reduced supply of photoassimilates to the coral host.

### Photosynthesis and respiration rates

Following the initial chlorophyll fluorescence measurements, fragments (*n* = 8) were transferred to eight individually temperature-controlled metabolic chambers (volume of 82 ml) to measure oxygen consumption in the dark (*R_dark_*) and production in the light (*Pn*). Chambers were filled with filtered seawater (0.2 μm) at the temperature of the sampling site. Chambers were then placed on magnetic stirrers, next to side-mounted custom-made fluorescent white LED lights emitting ca. 150 μmol photons m^−2^ s^−1^ directly to the surface of the chambers. Each jacketed chamber was equipped with a temperature probe connected to an Arduino Nano based controller and a water pump connected to the jacket. A warm water reservoir (40–45°C), heated with two 300-W heaters was used to control temperature in the chambers. When water in the chamber is below the set point in the Arduino, water from the reservoir flows into the chamber jacket and back to the reservoir using the water pumps. Using 10-second pumping intervals with 30-second intermissions, and injecting occasionally cold water (15–20°C) in the chamber jacket, the desired temperature ramping was reached without overshooting. Oxygen concentrations were measured with oxygen mini optrodes (FireStingO2, Pyroscience), with data logged at 1-second intervals using the Firesting Logger software (version 3.1).

### Symbiont cell density and surface measurement

Following the last chlorophyll fluorescence measurement, the fragments were incubated in 1 M NaOH at ambient temperature for several hours until the skeleton appeared completely white (i.e. full removal of the coral tissue; Zamoum and Furla, 2012). Only then, the symbiont cell densities were quantified from the bulk tissue solution using a hemacytometer and a digital microscope (Dino-Lite Edge AM4515T8, 900× magnification, DinoCapture 2.0 software). Fragment surface areas were estimated using the foil wrap method (Marsh, 1970). Briefly, aluminium foil was wrapped around each coral fragment, then stretched and photographed. The surface area of aluminium covering the coral skeleton was quantified using ImageJ1 (version 1.8.0).

### Data analysis

Relative percent change between the baseline and maximum temperature treatments was calculated for all photophysiological parameters (*F_v_/F_m_*, *rETR_max_*, *iK*, *alpha*, *NPQ/4_max_*), for each species, at each location. Rates of oxygen evolution of *R_dark_* and *Pn* were converted into concentrations of dissolved oxygen, given the specific salinity and temperature of the seawater used during the analysis (40‰ salinity in the GoA and 35‰ in the GoT in the winter; Ramsing and Gundersen, 1994; Youssouf *et al*., 2016; Cowburn *et al*., 2019). Gross photosynthesis (*Pg*), the amount of oxygen produced in the light after accounting for respiratory consumption, was derived from the equation *Pn* (light) = *Pg* (light) − *R_dark_* (dark), assuming a negligible difference between coral respiration in the light and dark. *R_dark_*, *Pg* and symbiont cell density were normalized to the surface area of each respective fragment.

Data analysis was performed using the statistical software R (version 3.6.2). All results are summarized in tables in the Supplementary section (Supplementary Tables S1–S16). Photophysiological data were analysed with paired Student or Wilcoxon rank sum tests for each species between temperature treatments at each location (Supplementary Table S2), and with Wilcoxon rank sum tests to compare the ambient treatments only between locations, for each species (Supplementary Table S3). Relative changes of each parameter were analysed using one-way ANOVA or Kruskal–Wallis rank sum tests (in case of heteroscedasticity) and TukeyHSD or Dunn’s post hoc tests, respectively (Supplementary Table S4). Differences between location of *YII*, *rETR*, fluorescence (*F*) and *NPQ*/4 at each PAR of the RLC were determined with Wilcoxon rank sum tests, at the specific local ambient temperature only (Supplementary Table S5). *R_dark_* and *Pg* were compared between locations for each species at each temperature using repeated-measures ANOVA, using ‘location’ and ‘temperature’ as fixed factors (Supplementary Tables S6 and S7). If significant, pairwise *t*-test post hoc analyses were conducted, with Bonferroni corrections for multiple comparisons (Supplementary Tables S8 and S9). The TPC of each individual fragment was fitted to Gaussian equation (Jurriaans and Hoogenboom, 2019) in order to derive *T_opt_* for each individual (Lynch and Gabriel, 1987; Padfield *et al*., 2021). Nonlinear least squares regression was used to determine the best fit to each TPC using the R package nls.multstart, as described in Aichelman *et al*. (2019). The uncertainty in the Gaussian fit and *T_opt_* was quantified using parametric bootstrapping (Padfield *et al*., 2021). Only the *T_opt_* for which the respective Gaussian fit was significant were used for downstream analysis. *T_opt_* obtained from *R_dark_* and *Pg* were compared using Wilcoxon tests between location, for each species (Table 1). These values were also compared to their respective local MMM SSTs using one-sample Wilcoxon test (Supplementary Table S14). *T_opt_* were compared between species for each location with Kruskal–Wallis tests (Supplementary Table S15 and S16).

**Table 1:** Summary statistics of the Wilcoxon tests performed on corals’ thermal optima (*T_opt_*) derived from gross photosynthetic and dark respiration TPCs

				Mean *T_opt_* (°C)	
	Species	W	p-values	GoA	GoT
Gross photosynthetic rate	*A. muricata*	15	0.083	28.9 ± 2.8	30.9 ± 0.5
	*M. dichotoma*	-	-	27.1 ± 0.9	-
	*P. verrucosa*	19	0.18	28.1 ± 0.8	28.7 ± 0.5
	*P. lobata*	4	0.126	28.4 ± 0.6	30.9 ± 2.4
	*S. hystrix*	8	1	29.0 ± 1.9	30.0 ± 0.3
	*S. pistillata*	0	**0.002**	28.3 ± 0.2	30.4 ± 0.7
Dark respiration rate	*A. muricata*	28	1	31.6 ± 2.1	31.4 ± 0.6
	*M. dichotoma*	-	-	32.4 ± 1.4	-
	*P. verrucosa*	-	-	32.5 ± 1.6	-
	*P. lobata*	8	0.2	29.9 ± 0.5	31.8 ± 2.5
	*S. hystrix*	-	-	-	31.0 ± 0.2
	*S. pistillata*	-	-	29.9 ± 1.3	-

The test statistics (W) and the *P*-values are indicated for each coral species. In bold, significant *P*-values (α = 0.05).

Lastly, symbiont cell density data were analysed using two-way ANOVA and Tukey HSD post hoc tests (Supplementary Table S4). Homogeneity of variances and data normality were checked using Levene’s and Shapiro–Wilk’s tests, respectively. In all cases, the significance level adopted was 95% (α = 0.05).

In order to integrate all the non-redundant physiological response variables from the thermal stress test (*F_v_/F_m_*, *rETR_max_*, *NPQ/4_max_*, *R_dark_*, *Pg*), principal components analysis (PCAs) were performed in R using the function ‘prcomp’ (Holland, 2019; Jurischka *et al*., 2020) based on a correlation matrix (normalized data), with location and temperature treatments (local ambient temperature and 34°C) included as fixed factors for each coral species. Component scores for each species are reported in Supplementary Table S10. In order to test the significance of the clustering, a permutational multivariate ANOVA (PERMANOVA) was conducted on Euclidian distances, with 999 permutations used to generate *P*-values and ‘location’ and ‘temperature treatments’ as fixed factors (Supplementary Table S11). PERMANOVAs were conducted using the ‘vegan’ package (Oksanen *et al*., 2018). Post hoc pairwise comparisons were performed using pairwise permutational MANOVAs (Supplementary Table S12).

**Figure 2:**
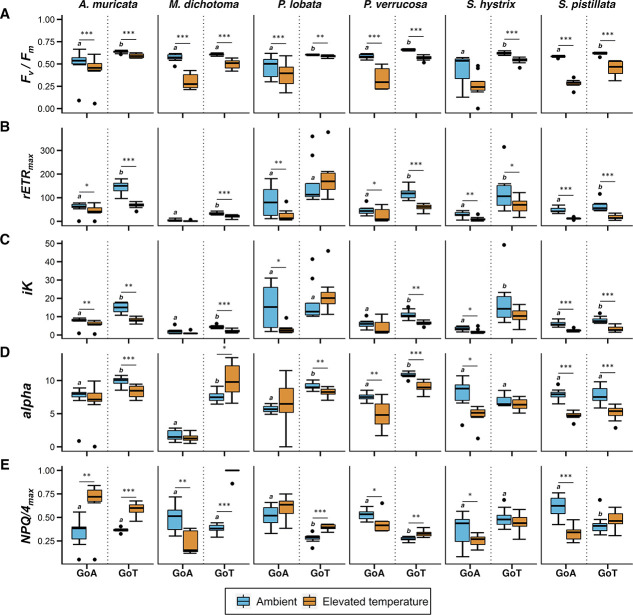
Dark-adapted *Fv/Fm*, *rETRmax*, *iK, alpha*, *NPQ/4max* of six reef-building coral species from the GoA or GoT, under ambient (22°C and 28°C, respectively) and then after elevation of temperatures (34°C). Asterisks represent significance levels from paired *t*-test or Wilcoxon rank sum test per species and location, between thermal treatments. *P <  0.05, **P < 0.01, ***P < 0.001. Different letters above the box in the ambient treatment indicate significant differences between location per species under ambient temperature (*n* = 8, α = 0.05). Black dots correspond to plots outliers. Error bars represent standard deviation.

**Figure 3:**
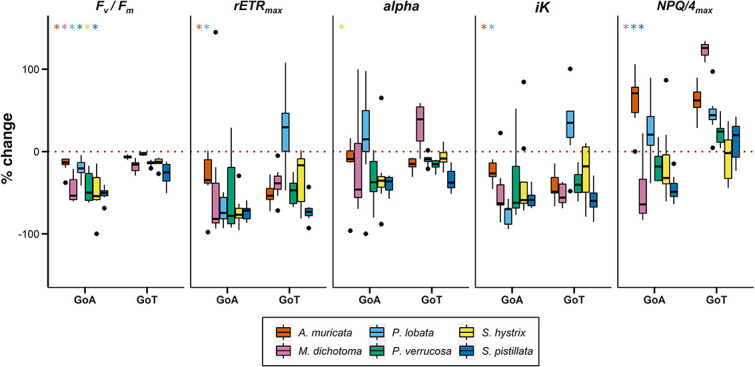
Percent change of dark-adapted *Fv/Fm*, *rETRmax*, *iK, alpha* and *NPQ/4max* of six reef-building coral species from the GoA or GoT, between ambient (22°C and 28°C, respectively) and elevated temperature (34°C) conditions. Asterisks represent significant differences from one-way ANOVA or Kruskal–Wallis tests between locations for each species (*n* = 8, α =0.05). The colour of the asterisks refers to the different species studied. Black dots correspond to plots outliers. Error bars represent standard deviation.

## Results

### Photophysiological performance

Rapid elevation of temperatures resulted in an overall decrease of photophysiological performance, varying as a function of species and sampling location (Fig. 2). *Millepora dichotoma*, *P. verrucosa* and *S. pistillata* showed a large decrease of *F_v_/F_m_* in both locations (*P* < 0.001; Figs 2A and 3; Supplementary Tables S2 and S4). Similarly, *rETR_max_* and *iK* decreased significantly for *P. lobata*, *S. hystrix* and *S. pistillata* in the GoA (*P* < 0.05) and for *A. muricata*, *M. dichotoma*, *P. verrucosa* and *S. pistillata* in the GoT (*P* < 0.001; Figs 2B,C and 3; Supplementary Tables S2 and S4). *NPQ/4_max_* and *alpha* revealed contrasting responses between the two locations (Figs 2D,E and 3; Supplementary Tables S2 and S4). Finally, the values of all parameters under ambient local temperatures, except from *NPQ/4_max_*, were 30–50% higher in the GoT compared to the GoA for all species except *S. pistillata* (*P* < 0.01; Fig. 2; Supplementary Table S3). The RLCs of *YII* and *rETR* support these observations, over the spectrum of PARs tested (Supplementary Figs S1 and S3; Supplementary Table S5). Interestingly, the RLCs of *NPQ/4* revealed an opposite trend for half of the species (*P. verrucosa*, *P. lobata* and *S. pistillata*; Supplementary Fig. S4; Supplementary Table S5), with higher values in the GoA than in the GoT.

**Figure 4:**
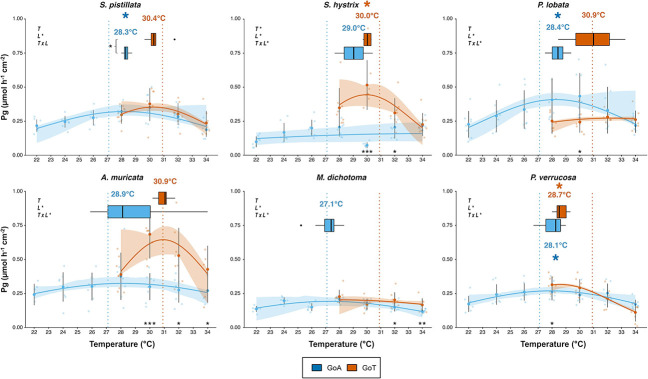
Gross photosynthesis (*Pg*) TPCs and derived thermal optima (*Topt*) of six coral species from GoA or GoT, between local ambient temperatures (22°C and 28°C, respectively) and 34°C (2°C above the summer maximum in the GoT). Fit lines are predictions of *Pg* obtained from Gaussian models, and confidence intervals are based on a non-parametric bootstrapping approach. Boxplots correspond to *Topt* derived from fitted models for each coral fragment. Temperatures above each box correspond to the average *Topt* (see also Table 1). Bold points represent means while transparent points correspond the raw Pg data. Dotted vertical lines indicate the MMM seawater temperatures in the GoA (27.1°C, blue) and in the GoT (30.9°C, orange). Results of repeated-measure ANOVAs are reported for each species using temperature (*T*) and location (*L*) as fixed factors and computing their interaction (*T x L*). Asterisks below the curves represent levels of significance of the post hoc pairwise *t*-test performed for each species between locations at each common temperature. Finally, asterisks to the left of the boxplots represent significant levels from Wilcoxon tests. ^*^P <  0.05, ^**^P < 0.01, ^***^P < 0.001 (*n* = 8, α = 0.05). Black dots correspond to plots outliers. Error bars represent 95% confidence intervals.

**Figure 5:**
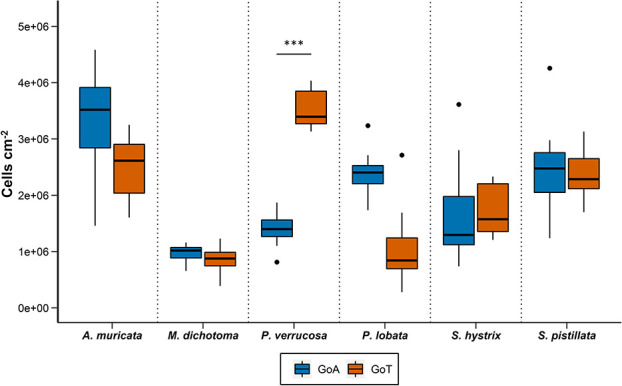
Symbiont cell density of six reef-building corals species from the GoA or GoT. Asterisks symbols represent significance differences from two-way ANOVA between location for each species at ambient temperature (*n* = 8, α = 0.05). ^*^P <  0.05, ^**^P < 0.01, ^***^P < 0.001. Black dots correspond to plots outliers. Error bars represent standard deviation.

**Figure 6:**
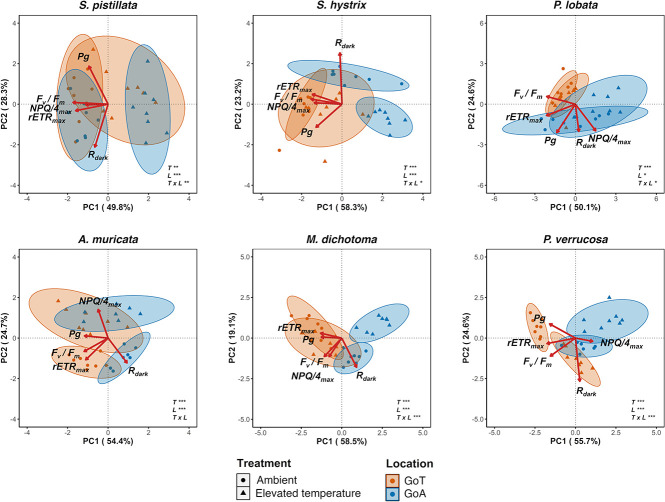
PCA ordination biplots using biological markers [*Fv/Fm*, *rETRmax*, *NPQ/4max*, dark respiration (*Rdark*), gross photosynthesis (*Pg*)], temperature [ambient (22°C in the GoA and 28°C in the GoT) and elevated temperature (34°C)] and location (GoA or GoT) for each coral species. Ellipses represent statistical clusters of 95% similarities. Red arrows represent eigenvectors for each biological marker used in this analysis. Results of PERMANOVA analysis are reported for each biplot using temperature (*T*) and location (*L*) as fixed factors and computing their interaction (T × L) (*n* = 8, α = 0.05).

### **TPCs, *T***_***opt***_  **and symbiont cell density**

Gaussian models were fitted for all coral species in both locations, for both *Pg* and *R_dark_*. The rate of *Pg* of *S. hystrix* at 32°C dropped unexpectedly before rising again at 34°C. After testing for the relevance of removing this data point, we decided to keep it in order to maintain the integrity of the dataset for this species. *T_opt_* were derived successfully, except for *M. dichotoma* in the GoT for *Pg* and *R_dark_* (Fig. 4) and for *S. pistillata*, *P. verrucosa* (GoT) and *S. hystrix* (GoA) for *R_dark_* only (Supplementary Fig. S5). The TPCs derived from *R_dark_* (Supplementary Fig. S5) revealed that all coral species except *S. hystrix* displayed *T_opt_* significantly higher than local MMM in the GoA (27.1°C; *P* < 0.05; Figs 4 and S5; Table 1 and Supplementary Table S13). TPCs derived from *Pg* (Fig. 4) showed a similar pattern, yet significant for three species only (*S. pistillata*, *P. lobata*, *P. verrucosa*; *P* < 0.05; Table 1 and Supplementary Table S13). Interestingly, for GoT corals, *T_opt_* derived from TPCs based on *Pg* were found significantly lower than local MMM (30.9°C) for *S. hystrix* and *P. verrucosa* only (*P* < 0.05; Fig. 4; Tables 1 and S13). Only *S. pistillata* displayed significant lower *T_opt_* in the GoA (28.3 ± 0.2°C) compared to the GoT (30.4 ± 0.7°C; *P* < 0.01; Fig. 4; Table 1). Differences in *T_opt_* between the GoA and the GoT were not significant for the other species. For most species, *R_dark_* increased with temperature (Supplementary Fig. S5), while *Pg* decreased after reaching a maximum rate at *T_opt_* (Fig. 4).

Symbiont cell densities were significantly different between locations for *P. verrucosa* only (*P* < 0.001; Fig. 5; Supplementary Table S4), with densities 3.1 times lower in the GoA. *Acropora muricata* and *S. pistillata* showed the highest symbiont densities in both locations, while the hydrozoan *M. dichotoma* had the lowest densities.

### Principal component analysis

The PCA performed for each species revealed different clustering patterns (Fig. 6) and explained 78.1%, 81.5%, 74.7%, 79.1%, 77.6% and 80.3% of the total variance for *S. pistillata*, *S. hystrix*, *P. lobata*, *A. muricata*, *M. dichotoma* and *P. verrucosa*, respectively. *F_v_/F_m_* contributed the most to PC1 for *S. pistillata*, *A. muricata* and *S. hystrix* and *rETR_max_* for *P. lobata*, *M. dichotoma* and *P. verrucosa* (Supplementary Table S10). Clustering appeared between temperature treatments in the GoA for *S. pistillata*, *S. hystrix*, *A. muricata* and *M. dichotoma* and for *A. muricata* and *P. verrucosa* in the GoT. The PERMANOVA yielded significant effects of locations and temperature treatments on the coral physiology and a significant interaction of these factors for all species except for *A. muricata* (*P* < 0.01; Supplementary Table S11). There were significant pairwise differences in coral response between locations and among temperature treatments for all species (*P* < 0.01; Supplementary Table S12).

## Discussion

Characterized by strong latitudinal environmental gradients, the Red Sea constitutes an ideal ‘natural laboratory’ to assess the capacity for thermal adaptation of corals. We provide the first comparison of coral temperature optima at both extremes of the Red Sea latitudinal gradient within a single study, spanning just 2 weeks, minimizing the likelihood of confounding effects, such as seasonality. Our results indicate that (i) symbiont cell densities are similar between the two locations, (ii) photophysiological performances vary between species and locations at local ambient temperatures, (iii) *T_opt_* is similar among locations for all species except *S. pistillata* and (iv) all species in the GoA live at temperatures below their *T_opt_* and close or above it in the GoT. Here we propose some testable hypotheses regarding these patterns.

### Conserved symbiont cell density between locations

Corals often display high variability in their algal symbiont densities both between and within species (Madin *et al*., 2016), which may constitute an adaptative mechanism to resist temperature variability (Fitt *et al*., 2000; Scheufen *et al*., 2017). Symbiont densities were not significantly different between the GoA and GoT, for all species investigated in the present study, except *P. verrucosa*. Since algal symbiont population sizes is primarily regulated by nutrient availability (Falkowski *et al*., 1993; Jones and Yellowlees, 1997) as a result of nutrient limitation (Cook and D’Elia, 1987; Krueger *et al*., 2020; Radecker *et al*., 2015), this finding is surprising given that nutrient concentrations in the GoT are approximately 15-fold higher than in the GoA (Sawall and Al-sofyani, 2015). Considering the high chlorophyll *a* levels in surface waters in the southern Red Sea (~4.0 mg m^−3^; Raitsos *et al*., 2013; Sawall *et al*., 2014), one explanation may be that the nutrients in the GoT are quickly consumed by phytoplankton, preventing coral algal symbionts from up-taking high nitrogen concentrations and propagating. For *P. verrucosa*, however, symbiont densities were 3.1 times higher in the GoT than in the GoA. This finding corroborates with previous work that reported lower cell densities for this species in the GoA (Maqna, Saudi Arabia), compared to the Farasan Island in the southern Red Sea, where nutrient concentrations were also higher relative to the GoA, particularly in winter (Sawall *et al*., 2014). The *Pocilloporidae* genus is characterized by a high level of gross morphological plasticity and shared morphological characteristics (Schmidt-Roach *et al*., 2014), which may render the identification of a given species, based on morphological traits, challenging. Therefore, molecular-based determination of the species identity should be used for future experiments to adress our finding and better understand this pattern. Comparing photosynthetic pigment concentrations (e.g. chlorophyll *a* and *c_2_*) to the winter symbiont cell density baselines established here may also assist in better elucidating the adaptative mechanisms at stake to resist the extreme summer seawater temperatures of the region. Moreover, since the higher chlorophyll *a* (primary productivity) and nutrient concentrations in the southern Red Sea are most pronounced during the winter (Raitsos *et al*., 2013), further research is needed to estimate the coral symbiont densities in the summer to determine a seasonal baseline for symbiont cell density and better understand local adaptation mechanisms to these environments with seasonally variable nutrient concentrations.

### Contrasting responses in photophysiological performances between locations at local ambient temperatures

Except for *S. pistillata*, all coral species consistently displayed values of *F_v_/F_m_*, *rETR_max_*, *iK* and *alpha* that were 30–50% higher in the GoT at local ambient temperatures, compared with the GoA, which may suggest higher efficiency of PSII in harvesting available light in the GoT during the winter time (Krueger *et al*., 2017). While comparisons of the raw fluorescence values (*F*) (Supplementary Fig. S3; Supplementary Table S5) under local ambient temperatures between the two locations showed no specific pattern, the *NPQ/4* showed significantly higher values in the GoA for half of the species tested (Supplementary Fig. S4; Supplementary Table S5). These coral species from the GoA may therefore dissipate excess light energy via NPQ (i.e. heat) pre-emptively at lower PARs compared to GoT corals, potentially as a photo-protective mechanism against high irradiances (~1200 μmol m^−2^ s^−1^ at the surface, 500–600 μmol m^−2^ s^−1^ at 5 m depths; Veal *et al*., 2010; Al-Rousan, 2012). Such patterns might also result from the differences in local ambient temperatures between the two locations. GoA corals, sampled in colder ambient conditions (22°C) compared to the GoT (28°C), may be more sensitive to higher irradiance at low temperature. Indeed, lower seawater temperatures have recently been shown to impair the photosynthetic efficiency of algal symbiont in GoAheat-tolerant corals (Bellworthy and Fine, 2021; Marangoni *et al*., 2021). RLCs at each temperature tested would help understand the temperature-specific response of the coral holobiont under increasing light irradiance.

### Temperature optima signatures between the GoA and the GoT

Adaptation and/or acclimatization of biological traits across an organism’s geographic range as a result of environmentally driven selection should result in population-specific variations in thermal performance (Angilletta, 2009b; Sanford and Kelly, 2011). No significant difference was found in the *T_opt_* (based on both *Pg* and *R_dark_* rates) of the different species tested between the two locations except for *S. pistillata*, which reveals an exceptional continuity in the physiological performances of these common reef-building species across a large latitudinal range. Together with the similarity in symbiont cell densities between the two locations, this finding supports the GoA coral refuge hypothesis, suggesting that the present GoA corals inherited their physiological performances from coral populations selected for their thermal resistance near the GoT during the successive re-colonization events of the Red Sea (Fine *et al*., 2013). Yet, quantifying the *T_opt_* of the same coral species in various Red Sea reefs located between the GoA and the GoT is needed to support this hypothesis. Additionally, seasonal acclimation of thermal performances was recently shown for two scleractinian corals species from the Great Barrier Reef, displaying either higher *T_opt_*, or a wider thermal breadth (Jurrians and Hoogenboom, 2020). As the present study was conducted in the wintertime, the response of the coral species used here should also be assessed under the same experimental conditions during the summertime, to detect a possible seasonal acclimation.

*Stylophora pistillata* is the only species that displayed significant lower *T_opt_* (based on *Pg*) in the GoA compared to the GoT (Fig. 4). Similarly, the Caribbean reef-building coral *O. franski* displayed lower *T_opt_* (based on *Pg*) in the cooler waters of Bermuda, compared to the warmer waters of Panama, and was suggested to have adapted to the local colder conditions (Silbiger *et al*., 2019). Moreover, *S. pistillata* systematically showed the strongest decrease in photophysiological performances as a result of elevation of temperatures (trend visible on Figs 2 and 3), but more replicates are needed to characterize this species as the most sensitive. A recent study reported that *S. pistillata* from the GoA may be living close to its cold-water bleaching threshold (Bellworthy and Fine, 2021), which together with our results may suggest that *S. pistillata* is going through a population-level selection (i.e. adaptation) to the cooler waters of the GoA and may be subsequently losing its high thermal resistance compared to other common reef-building species. This finding is particularly of importance as (i) *S. pistillata*, widely distributed across the Indo-Pacific region (Veron, 2000), is the most abundant coral of the shallow fraction of the northern GoA (10.6% of all species between 0 and30 m deep; Kramer *et al*., 2020) and (ii) it is commonly considered a ‘laboratory rat’ (Sawall and Al-sofyani, 2015), used extensively as a model organism in laboratory experiments simulating temperature stress (Banc-Prandi and Fine, 2019; Bellworthy and Fine, 2017; Krueger *et al*., 2017; Savary *et al*., 2021; Voolstra *et al*., 2020; Meziere *et al*., 2021). We therefore question the relevance of using this species in heat stress experiments in the future to assess the thermal resistance of coral species from the Red Sea.

A similar pattern of increasing *T_opt_* with increasing ambient temperature has already been reported in other organisms, such as macrophytes (Santamaria and van Vierssen 1997) and trees (tropical versus temperate; Cunningham and Read, 2002), but barely for reef-building scleractinians (Aichelman *et al*., 2019; Jurriaans and Hoogenboom, 2019). The pattern observed in the present study for *S. pistillata* is consistent with cogradient variation (CoGV), for which the warm population in the GoT exhibits elevated metabolic rates compared with the cold population in the GoA, across a temperature range (Angilletta, 2009b; Conover *et al*., 2009; Sanford and Kelly, 2011). CoGV typically occurs when variations of environmental conditions (in the present study, increasing temperatures across the Red Sea latitudinal range) and selection pressure (e.g. elevated temperature) act synergistically on a biological trait across a geographical range (Conover *et al*., 2009). CoGV was also reported for *S. pistillata* in the Red Sea, which displayed increasing thermal thresholds (based on measurements of *F_v_/F_m_*) across six sites with increasing MMMs (Evensen *et al*., *unpublished*). Conversely, studies using the same TPC approach on coral populations spanning latitudinal gradients along the eastern US coast and Great Barrier Reef did not find any evidence of CoGV (Aichelman *et al*., 2019; Jurriaans and Hoogenboom, 2019). Future research should assess the response of the present species from both locations in a common garden experiment, in order to validate the CoGV pattern observed in the present study.

### Species-specific successes in the local environment

As stated above, the *T_opt_* of all species were above the local MMM in the GoA (based on *R_dark_* rates, and supported by *Pg* for three of the species) and close to or below it in the GoT (Figs 4 and S5; Table 1 and Supplementary Table S13). Thermal thresholds (based on *F_v_/F_m_* measurements) relative to local MMMs were found to be higher in the northern Red Sea compared to the central and southern Red Sea for the same reef-building coral species studied herein (Evensen *et al*., *unpublished*; Savary *et al*., 2021). These findings support the hypothesis that coral populations from the northern Red Sea may not be experiencing warm-water bleaching in the next few decades as they live in suboptimal thermal conditions, far below their upper thermal threshold (Evensen *et al*., 2021; Fine *et al*., 2013; Krueger *et al*., 2017; Osman *et al*., 2018; Voolstra *et al*., 2020; Voolstra *et al*., 2021), yet closer to their cold-water bleaching threshold (Bellworthy and Fine, 2021). Additionally, our results support the findings of Krueger *et al*. (2017), as *S. pistillata* from the GoA displays improved physiological performances (*Pg* and *R_dark_* rates) at temperatures 1–2°C above the local MMM (27.1°C, for a *T_opt_* at 28.3 ± 0.2°C). Conversely, GoT corals may be living close to their upper thermal threshold and close or above their *T_opt_* (e.g. *P. verrucosa* and S*. hystrix*, based on *Pg*). Yet, GoT corals have not been experiencing mass bleaching in the past decade, despite rising seawater temperatures (Cowburn *et al*., 2019). This may be explained by the higher turbidity of the GoT waters. Multiple studies have highlighted the diversity and adaptive capacity of turbid-zone coral communities across large spatio-temporal ranges (Browne *et al*., 2010; Bull, 1982; Butler *et al*., 2013; Guest *et al*., 2016; Lafratta *et al*., 2017; Morgan *et al*., 2017; Richards *et al*., 2015). On the Great Barrier Reef, nearshore coral communities experienced only minor bleaching compared with offshore reefs under similar heat stress (Morgan *et al*., 2017). During long periods of thermal stress, suspended sediment and organic matter may attenuate UV radiation therefore alleviating radiative stress (van Woesik *et al*., 2012). Being light limited, corals in turbid environments are effectively combing phototrophic and heterotrophic feeding, including particulate organic matter (Anthony and Fabricius, 2000; Anthony *et al*., 2005), which can promote resistance to temperature-induced bleaching (Ferrier-Pagès *et al*., 2018; Houlbrèque and Ferrier-Pagès, 2009). Yet not all coral species are able to increase their heterotrophic feeding capacity (Grottoli *et al*., 2006), which may contribute to structuring coral communities in turbid environments, favouring higher abundances of more heterotrophic species (Done *et al*., 2007; Grottoli *et al*., 2006).

No clear ‘winners’ or ‘losers’ of the short heat stress could be identified based on the photophysiological performance. Yet, based on *F_v_/F_m_*, *A. muricata* and *P. lobata* seemed less affected by the rapid elevation of temperatures, displaying low relative change compared to the other species (Figs 2 and 3; Supplementary Tables S2 and S4). *Acropora* and *Porites* genus are known to be ‘moderately’ affected by thermal bleaching (Dalton *et al*., 2020; McClanahan *et al*., 2004) and identified as ‘winners’ of thermal stress (Loya *et al*., 2001; van Woesik *et al*., 2011). Their high thermal resistance (van Woesik *et al*., 2011) may promote their domination of reefs after thermal bleaching events, as described in the Persian/Arabian Gulf where *Porites* are found in higher frequency over the other corals (88% of coral cover; Burt *et al*., 2011). Yet, additional research is needed to confirm the pattern here obtained and support this hypothesis.

Overall, our study suggests a potentially complex interplay between local thermal conditions, nutrient concentrations and irradiance in shaping the temperature optima signature of reef-building coral species. Corals from the GoA may be currently living in suboptimal thermal conditions, pushing some species like *S. pistillata* to potentially undergo selection to the cooler waters of the northern Red Sea, a process that may ultimately result in the loss of its resistance to elevated temperatures in the long term. Conversely, GoT populations, living close or above their temperature optima and close to their upper thermal threshold all year-long, might be at risk during future summer extremes, a threat potentially mitigated by the high turbidity of the local reef waters. Such comparative work between contrasting environments yet overlapping species is increasingly needed to (i) determine the state of vulnerability of specific reefs to elevated SSTs, (ii) understand the environmental drivers of their susceptibility and (iii) examine the mechanisms of adaptation and acclimatization to local conditions, in order to guide conservation management.

## Funding

This work was supported by USA–Israel Binational Science Foundation (grant #2016403 to D.B. and M.F.).

## Data Availability Statement

The data underlying this article will be shared on reasonable request to the corresponding author.

## Author contributions

G.B.P and M.F. designed the experiment. G.B.P, M.F and G.P. collected the data. G.B.P analysed the data and, together with M.F, N.R.E, D.J.B, G.P and M.O.Y., interpreted the results. G.B.P and M.F wrote the manuscript with input from all co-authors.

## Supplementary Material

Supplementary_material_coac002
