# Supplementary material for: Assessment of temperature optimum signatures of corals at both latitudinal extremes of the Red Sea
Source: Conserv Physiol. 2022 Feb 18;10(1):coac002. doi: 10.1093/conphys/coac002 (PMC9040280; doi:10.1093/conphys/coac002)
Supplement: Supplementary_material_coac002 [file supplementary_material_coac002.docx]

# Supplementary material

**Table S1.** **GPS coordinates of the different sampling sites in the Gulf of Aqaba (GoA) and the Gulf of Tadjoura (GoT).** Numbers for each site refer to the symbols used on the Figure 1. Coordinates are expressed in decimal degrees.

| **Sampling sites** | **Species** | **Latitude** | **Longitude** |
| --- | --- | --- | --- |
| 1 - IUI nursery (Eilat, GoA) | all | N 29.50232 | E 34.91703 |
| 2 - Maskali sunken buoy (GoT) | *P. verrucosa* | N 11.69777 | E 43.14559 |
| 3 - Obock forereef (GoT) | *P. lobata* | N 11.95862 | E 43.31312 |
| 4 - Sable Blanc (GoT) | *S. pistillata* | N 11.77573 | E 42.92470 |
| 5 - Ghoubet Wall/Red Virgin (GoT) | *M. dichotoma* | N 11.51020 | E 42.67162 |
| 6 - Ras Korali (GoT) | *S. hystrix –*  *A. muricata* | N 11.57994 | E 42.79142 |

**Table S2. Summary statistics of the Student or the Wilcoxon rank sum (in italic) tests performed on the photophysiological data.** The degree of freedom (df), the t-values (V-values) as well as the p-values are indicated for each parameter, each coral and each location (GoA or GoT) examined between the ambient and elevated temperature treatments. In bold, significant p-values (n = 8, α = 0.05).

| **_Source of variation_** | | **_df_** | | | | | **_t-values/_*_V-values_*** | | | | | **_p-values_** | | | | |
| --- | --- | --- | --- | --- | --- | --- | --- | --- | --- | --- | --- | --- | --- | --- | --- | --- |
|  | | *F_v_/F_m_* | *rETR_max_* | *NPQ/4_max_* | *alpha* | *iK* | *F_v_/F_m_* | *rETR_max_* | *NPQ/4_max_* | *alpha* | *iK* | *F_v_/F_m_* | *rETR_max_* | *NPQ/4_max_* | *alpha* | *iK* |
| **GoA** | *S. pistillata* | 7 | 7 | 7 | 7 | 7 | 16.94 | 8.17 | 6.73 | 8.29 | 8.89 | **< 0.001** | **< 0.001** | **< 0.001** | **< 0.001** | **< 0.001** |
|  | *S. hystrix* | 7 | 7 | 7 | 7 | 7 | 1.99 | 3.40 | 2.48 | 2.85 | 2.62 | 0.087 | **0.0088** | **0.042** | **0.025** | **0.035** |
|  | *P. verrucosa* | 7 | 7 | 7 | 7 | 7 | 6.93 | 2.941 | 2.39 | 3.23 | 2.00 | **< 0.001** | **0.022** | **0.048** | **0.015** | 0.085 |
|  | *P. lobata* | 7 | 7 | 7 | 7 | 7 | 6.80 | 3.51 | -1.37 | -0.68 | 3.44 | **< 0.001** | **0.001** | 0.21 | 0.51 | **0.011** |
|  | *A. muricata* | 7 | 7 | 7 | *7* | 7 | 8.13 | 2.98 | -4.99 | *22.0* | 4.84 | **< 0.001** | **0.02** | **0.0016** | *0.64* | **0.0019** |
|  | *M. dichotoma* | 7 | 7 | 7 | 7 | 7 | 8.56 | 1.34 | 3.60 | 0.83 | 1.48 | **< 0.001** | 0.22 | **0.0087** | 0.43 | 0.18 |
| **GoT** | *S. pistillata* | 7 | 7 | 7 | 7 | 7 | 5.38 | 7.03 | -1.12 | 6.49 | 6.95 | **0.001** | **< 0.001** | 0.29 | **< 0.001** | **< 0.001** |
|  | *S. hystrix* | 7 | *7* | 7 | 7 | *7* | 6.07 | *35.0* | 0.93 | 1.79 | *28.0* | **< 0.001** | ***0.016*** | 0.38 | 0.12 | *0.19* |
|  | *P. verrucosa* | 7 | 7 | 7 | 7 | 7 | 12.34 | 5.76 | -4.41 | 7.41 | 4.89 | **< 0.001** | **< 0.001** | **0.0031** | **< 0.001** | **0.0018** |
|  | *P. lobata* | 7 | *7* | 7 | 7 | 7 | 5.02 | *8.0* | -7.23 | 4.05 | -1.16 | **0.0015** | *0.19* | **< 0.001** | **0.0049** | 0.29 |
|  | *A. muricata* | 7 | 7 | 7 | 7 | 7 | 8.14 | 7.22 | -9.37 | 5.46 | 5.34 | **< 0.001** | **< 0.001** | **< 0.001** | **< 0.001** | **0.0011** |
|  | *M. dichotoma* | 7 | 7 | 7 | 7 | 7 | 6.78 | 6.33 | -21.77 | -3.18 | 14.16 | **< 0.001** | **< 0.001** | **< 0.001** | **0.015** | **< 0.001** |

**Table S3. Summary statistics of the Wilcoxon rank sum tests performed on the photophysiological data for each species between the locations (GoA or GoT) in ambient temperature conditions only.** The p-values are indicated for each parameter. In bold, significant p-values (n = 8, α = 0.05).

| **_Source of variation_** | | **_p-values_** | | | | |
| --- | --- | --- | --- | --- | --- | --- |
|  | | *F_v_/F_m_* | *rETR_max_* | *NPQ/4_max_* | *alpha* | *iK* |
| **GoA - GoT** | *S. pistillata* | **0.0074** | **0.049** | **0.0086** | 0.79 | **0.038** |
|  | *S. hystrix* | **< 0.001** | **< 0.001** | 0.21 | 0.065 | **< 0.001** |
|  | *P. verrucosa* | **< 0.001** | **< 0.001** | **< 0.001** | **< 0.001** | **< 0.001** |
|  | *P. lobata* | **0.013** | 0.28 | **< 0.001** | **< 0.001** | 0.51 |
|  | *A. muricata* | **0.024** | **< 0.001** | 0.71 | **< 0.001** | **< 0.001** |
|  | *M. dichotoma* | 0.093 | **< 0.001** | 0.16 | **< 0.001** | **0.0069** |

**Table S4. Summary statistics of the tests performed on the relative change of the photophysiological data (One-Way ANOVAs or Kruskal Wallis rank sum test (in italic)) and symbiont cell density (Two-Ways ANOVAs).** The degree of freedom (df), the sum square (SS), the F-values as well as the p-values are indicated for each parameter, and each location. In bold, significant p-values (n = 8, α = 0.05).

| **_Source of variation_** | | **_df_** | **_SS_** | **_F-value /_ *_chi-squared_*** | **_p-values_** |
| --- | --- | --- | --- | --- | --- |
| **Between species in the GoA** | % *F_v_/F_m_* | *5* | *-* | *20.11* | ***0.0012*** |
|  | % *rETR_max_* | *5* | *-* | *4.57* | *0.47* |
|  | % *NPQ/4_max_* | *5* | *-* | *27.68* | ***< 0.001*** |
|  | % *alpha* | *5* | *-* | *8.81* | *0.12* |
|  | % *iK* | *5* | *-* | *16.56* | ***0.0054*** |
| **Between species in the GoT** | *% F_v_/F_m_* | *5* | *-* | *33.76* | ***< 0.001*** |
|  | *% rETR_max_* | *5* | *-* | *24.03* | ***< 0.001*** |
|  | *% NPQ/4_max_* | *5* | *144477* | *38.09* | ***< 0.001*** |
|  | *% alpha* | *5* | *-* | *28.78* | ***< 0.001*** |
|  | *% iK* | *5* | *-* | *21.32* | ***< 0.001*** |
| **Between locations for *S. pistillata*** | *% F_v_/F_m_* | *-* | *-* | *5.0* | ***0.0029*** |
|  | *% rETR_max_* | *-* | *-* | *30.0* | *0.88* |
|  | *% NPQ/4_max_* | *-* | *-* | *1.0* | **< 0.001** |
|  | *% alpha* | *-* | *-* | *26.0* | *0.57* |
|  | *% iK* | *-* | *-* | *33.0* | *0.96* |
| **Between locations for *A. muricata*** | *% F_v_/F_m_* | *-* | *-* | *6.0* | ***0.0047*** |
|  | *% rETR_max_* | *-* | *-* | *51.0* | ***0.049*** |
|  | *% NPQ/4_max_* | *-* | *-* | *42.0* | *0.33* |
|  | *% alpha* | *-* | *-* | *45.0* | *0.19* |
|  | *% iK* | *-* | *-* | *51.0* | ***0.049*** |
| **Between locations for *S. hystrix*** | *% F_v_/F_m_* | *-* | *-* | *11.0* | ***0.028*** |
|  | *% rETR_max_* | *-* | *-* | *18.0* | *0.16* |
|  | *% NPQ/4_max_* | *-* | *-* | *24.0* | *0.44* |
|  | *% alpha* | *-* | *-* | *8.0* | ***0.0104*** |
|  | *% iK* | *-* | *-* | *25.0* | *0.51* |
| **Between locations for *P. verrucosa*** | *% F_v_/F_m_* | *-* | *-* | *3.0* | ***0.0011*** |
|  | *% rETR_max_* | *-* | *-* | *24.0* | *0.44* |
|  | *% NPQ/4_max_* | *-* | *-* | *3.0* | ***0.0011*** |
|  | *% alpha* | *-* | *-* | *20.0* | *0.26* |
|  | *% iK* | *-* | *-* | *23.0* | *0.38* |
| **Between locations for *P. lobata*** | *% F_v_/F_m_* | *-* | *-* | *2.0* | **< 0.001** |
|  | *% rETR_max_* | *-* | *-* | *0* | **< 0.001** |
|  | *% NPQ/4_max_* | *-* | *-* | *18.0* | *0.16* |
|  | *% alpha* | *-* | *-* | *50.0* | *0.065* |
|  | *% iK* | *-* | *-* | *0* | **< 0.001** |
| **Between locations for *M. dichotoma*** | *% F_v_/F_m_* | *-* | *-* | *3.0* | ***0.0011*** |
|  | *% rETR_max_* | *-* | *-* | *24.0* | *0.44* |
|  | *% NPQ/4_max_* | *-* | *-* | *0* | **< 0.001** |
|  | *% alpha* | *-* | *-* | *23.0* | *0.38* |
|  | *% iK* | *-* | *-* | *35.0* | *0.79* |
| **Symbiont cell density** | Species | 5 | 4.620e^13^ | 9.88 | **< 0.001** |
|  | Sampling site | 1 | 8.122e^11^ | 0.87 | 0.35 |
|  | Species * Sampling site | 5 | 4.379e^13^ | 9.37 | **< 0.001** |

**Table S5. Summary statistics (p-values) of the Wilcoxon tests on the RLC data (*F*, *YII*, *rETR*, *NPQ/4*) to assess the difference between the GoA and the GoT under ambient temperatures for each coral species.** In bold, significant p-values (n = 8, α = 0.05).

|  |  | **PAR (µmol quanta m^-2^ s^-1^)** | | | | | | | | | | | | |
| --- | --- | --- | --- | --- | --- | --- | --- | --- | --- | --- | --- | --- | --- | --- |
| **Species** | **Parameter** | **0** | **1** | **21** | **56** | **111** | **186** | **281** | **336** | **396** | **461** | **531** | **611** | **701** |
| ***A. muricata*** | ***F*** | **0.038** | **0.038** | 0.270 | **0.021** | 0.141 | 0.279 | 0.442 | 0.328 | 0.328 | 0.279 | 0.505 | 0.495 | 0.721 |
|  | ***YII*** | **0.024** | **0.021** | 0.234 | **0.003** | **0.004** | **< 0.001** | **< 0.001** | **< 0.001** | **< 0.001** | **< 0.001** | **< 0.001** | **< 0.001** | **< 0.001** |
|  | ***rETR*** | NA | **0.021** | 0.234 | **0.003** | **0.004** | **< 0.001** | **< 0.001** | **< 0.001** | **< 0.001** | **< 0.001** | **0.000** | **0.001** | **< 0.001** |
|  | ***NPQ/4*** | NA | 0.075 | **0.032** | 0.957 | 0.558 | 0.874 | 1.000 | 0.752 | 0.598 | 0.635 | 0.752 | 0.833 | 0.713 |
| ***M. dichotoma*** | ***F*** | **0.005** | **< 0.001** | **< 0.001** | **< 0.001** | **0.006** | **0.007** | **0.015** | 0.083 | 0.161 | 0.382 | 0.645 | 0.878 | 0.878 |
|  | ***YII*** | 0.093 | **< 0.001** | **< 0.001** | **< 0.001** | **< 0.001** | **< 0.001** | **< 0.001** | **< 0.001** | **< 0.001** | **< 0.001** | **< 0.001** | **< 0.001** | **< 0.001** |
|  | ***rETR*** | NA | **< 0.001** | **< 0.001** | **< 0.001** | **< 0.001** | **< 0.001** | **< 0.001** | **< 0.001** | **< 0.001** | **< 0.001** | **< 0.001** | **< 0.001** | **< 0.001** |
|  | ***NPQ/4*** | NA | 0.075 | **0.009** | **0.012** | **0.015** | 0.155 | 0.291 | 0.318 | 0.226 | 0.172 | 0.207 | 0.248 | 0.161 |
| ***P. verrucosa*** | ***F*** | **0.021** | **0.028** | 0.093 | 0.279 | 0.161 | **< 0.001** | **< 0.001** | **< 0.001** | **< 0.001** | **< 0.001** | **< 0.001** | **< 0.001** | **< 0.001** |
|  | ***YII*** | **< 0.001** | **< 0.001** | **< 0.001** | **< 0.001** | **< 0.001** | **< 0.001** | **< 0.001** | **< 0.001** | **< 0.001** | **< 0.001** | **< 0.001** | **< 0.001** | **< 0.001** |
|  | ***rETR*** | NA | **< 0.001** | **< 0.001** | **< 0.001** | **< 0.001** | **< 0.001** | **< 0.001** | **< 0.001** | **< 0.001** | **< 0.001** | **< 0.001** | **< 0.001** | **< 0.001** |
|  | ***NPQ/4*** | NA | NA | **< 0.001** | **< 0.001** | **< 0.001** | **< 0.001** | **< 0.001** | **< 0.001** | **< 0.001** | **< 0.001** | **< 0.001** | **< 0.001** | **< 0.001** |
| ***P. lobata*** | ***F*** | **< 0.001** | **< 0.001** | **< 0.001** | **< 0.001** | **< 0.001** | **< 0.001** | **< 0.001** | **< 0.001** | **< 0.001** | **< 0.001** | **< 0.001** | **< 0.001** | **< 0.001** |
|  | ***YII*** | **0.013** | **0.016** | **< 0.001** | **< 0.001** | **< 0.001** | **< 0.001** | **< 0.001** | **< 0.001** | **< 0.001** | **< 0.001** | **< 0.001** | **< 0.001** | **< 0.001** |
|  | ***rETR*** | NA | **< 0.001** | **< 0.001** | **< 0.001** | **< 0.001** | **< 0.001** | **< 0.001** | **< 0.001** | **< 0.001** | **< 0.001** | **< 0.001** | **< 0.001** | **< 0.001** |
|  | ***NPQ/4*** | NA | 0.382 | NA | **0.004** | **< 0.001** | **< 0.001** | **< 0.001** | **< 0.001** | **< 0.001** | **< 0.001** | **< 0.001** | **< 0.001** | **< 0.001** |
| ***S. hystrix*** | ***F*** | **0.021** | 0.083 | 0.442 | 0.105 | 0.130 | 0.234 | 0.279 | 0.574 | 0.878 | 0.721 | 0.645 | 0.372 | 0.318 |
|  | ***YII*** | **< 0.001** | **< 0.001** | 0.172 | 0.959 | 0.442 | **0.046** | **0.050** | **0.005** | **0.012** | **< 0.001** | **0.015** | **< 0.001** | **< 0.001** |
|  | ***rETR*** | NA | **< 0.001** | 0.172 | 0.959 | 0.442 | **0.046** | **0.050** | **0.005** | **0.012** | **< 0.001** | **0.015** | **< 0.001** | **< 0.001** |
|  | ***NPQ/4*** | NA | 0.587 | 0.154 | 0.155 | 0.269 | 0.462 | 0.798 | 0.959 | 1.000 | 0.878 | 0.574 | 0.442 | 0.207 |
| ***S. pistillata*** | ***F*** | **0.006** | **0.003** | **0.015** | 0.105 | 0.574 | 0.798 | 0.400 | 0.442 | 0.328 | 0.195 | 0.161 | 0.074 | 0.103 |
|  | ***YII*** | **0.007** | **0.014** | 0.189 | 0.382 | 0.599 | 0.878 | 0.833 | 0.372 | 0.065 | 0.103 | 0.092 | 0.793 | **0.031** |
|  | ***rETR*** | NA | **0.014** | 0.189 | 0.382 | 0.599 | 0.878 | 0.833 | 0.372 | 0.065 | 0.103 | 0.092 | 0.793 | **0.031** |
|  | ***NPQ/4*** | NA | NA | 0.425 | 0.317 | 0.092 | **0.028** | **0.018** | 0.013 | **0.010** | **0.012** | **0.010** | **0.010** | **0.009** |

**Table S6. Summary statistics of the repeated-measures ANOVA tests performed on gross photosynthetic rates.** The F-statistic values (F), the generalized effect size (ges), and the p-values are indicated for each coral species using “Location” and “Temperature” as fixed factors. In bold, significant p-values (n = 8, α = 0.05).

| **Species** | **Source of variation** | **F** | **ges** | **p-value** |
| --- | --- | --- | --- | --- |
| ***S. pistillata*** | **Location** | 0.18 | 0.01 | 0.681 |
|  | **Temperature** | 28.12 | 0.35 | **< 0.001** |
|  | **Location*Temperature** | 1.60 | 0.03 | 0.22 |
| ***A.muricata*** | **Location** | 9.70 | 0.35 | **0.017** |
|  | **Temperature** | 7.73 | 0.13 | **0.001** |
|  | **Location*Temperature** | 30.69 | 0.20 | **< 0.001** |
| ***S. hystrix*** | **Location** | 12.52 | 0.45 | **0.009** |
|  | **Temperature** | 5.71 | 0.07 | **0.005** |
|  | **Location*Temperature** | 58.77 | 0.41 | **< 0.001** |
| ***M. dichotoma*** | **Location** | 4.96 | 0.13 | 0.061 |
|  | **Temperature** | 23.16 | 0.34 | **< 0.001** |
|  | **Location*Temperature** | 7.89 | 0.1 | **0.001** |
| ***P. verrucosa*** | **Location** | 0.004 | 0.0001 | 0.952 |
|  | **Temperature** | 50.75 | 0.49 | **< 0.001** |
|  | **Location*Temperature** | 11.96 | 0.16 | **< 0.001** |
| ***P. lobata*** | **Location** | 2.39 | 0.14 | 0.166 |
|  | **Temperature** | 6.94 | 0.11 | **0.017** |
|  | **Location*Temperature** | 16.39 | 0.15 | **0.001** |

**Table S7. Summary statistics of the repeated-measures ANOVA tests performed on dark respiration rates.** The F-statistic values (F), the generalized effect size (ges), and the p-values are indicated for each coral species using “Location” and “Temperature” as fixed factors. In bold, significant p-values (n = 8, α = 0.05).

| **Species** | **Source of variation** | **F** | **ges** | **p-value** |
| --- | --- | --- | --- | --- |
| ***S. pistillata*** | **Location** | 0.017 | 0.001 | 0.899 |
|  | **Temperature** | 1.842 | 0.05 | 0.209 |
|  | **Location*Temperature** | 1.5 | 0.053 | 0.262 |
| ***A. muricata*** | **Location** | 2.679 | 0.147 | 0.146 |
|  | **Temperature** | 1.67 | 0.067 | 0.234 |
|  | **Location*Temperature** | 16.851 | 0.192 | **0.001** |
| ***S. hystrix*** | **Location** | 10.415 | 0.402 | **0.015** |
|  | **Temperature** | 23.918 | 0.234 | **< 0.001** |
|  | **Location*Temperature** | 84.736 | 0.496 | **< 0.001** |
| ***M. dichotoma*** | **Location** | 2.511 | 0.041 | 0.157 |
|  | **Temperature** | 3.022 | 0.058 | 0.052 |
|  | **Location*Temperature** | 6.127 | 0.175 | **0.004** |
| ***P. verrucosa*** | **Location** | 60.924 | 0.593 | **< 0.001** |
|  | **Temperature** | 16.954 | 0.361 | **< 0.001** |
|  | **Location*Temperature** | 34.342 | 0.543 | **< 0.001** |
| ***P. lobata*** | **Location** | 0.143 | 0.01 | 0.716 |
|  | **Temperature** | 1.942 | 0.069 | 0.199 |
|  | **Location*Temperature** | 0.623 | 0.016 | 0.514 |

**Table S8. Summary statistics of the pairwise t-tests post-hoc analysis of the repeated-measures ANOVA tests, performed on gross photosynthesic rates.** The statistics values, the degree of freedom (df), and the p-values are indicated for each coral species between locations (GoA and GoT for Gulf of Aqaba and Gulf of Tadjoura) at each temperature. In bold, significant p-values (n = 8, α = 0.05).

| **Species** | **Temperature** | **statistics** | **df** | **adjusted p-values** |
| --- | --- | --- | --- | --- |
| ***S. pistillata*** | 28 | 0.736 | 7 | 0.486 |
|  | 30 | -0.415 | 7 | 0.691 |
|  | 32 | -0.443 | 7 | 0.671 |
|  | 34 | -1.175 | 7 | 0.278 |
| ***A. muricata*** | 28 | -0.157 | 7 | 0.88 |
|  | 30 | -7.209 | 7 | **< 0.001** |
|  | 32 | -3.456 | 7 | **0.011** |
|  | 34 | -2.560 | 7 | **0.038** |
| ***S. hystrix*** | 28 | -2.254 | 7 | 0.059 |
|  | 30 | -6.676 | 7 | **< 0.001** |
|  | 32 | -2.377 | 7 | **0.049** |
|  | 34 | -0.205 | 7 | 0.843 |
| ***M. dichotoma*** | 28 | -1.177 | 7 | 0.278 |
|  | 30 | 0.604 | 7 | 0.565 |
|  | 32 | -3.362 | 7 | **0.012** |
|  | 34 | -2.829 | 7 | **0.025** |
| ***P. verrucosa*** | 28 | -2.406 | 7 | **0.047** |
|  | 30 | -1.808 | 7 | 0.114 |
|  | 32 | 1.255 | 7 | 0.25 |
|  | 34 | 2.277 | 7 | 0.057 |
| ***P. lobata*** | 28 | 2.118 | 7 | 0.072 |
|  | 30 | 2.987 | 7 | **0.02** |
|  | 32 | 0.771 | 7 | 0.466 |
|  | 34 | -1.110 | 7 | 0.304 |

**Table S9. Summary statistics of the pairwise t-tests post-hoc analysis of the repeated-measures ANOVA tests, performed on dark respiration rates.** The statistics values, the degree of freedom (df), and the p-values are indicated for each coral species between locations (GoA and GoT for Gulf of Aqaba and Gulf of Tadjoura) at each temperature. In bold, significant p-values (n = 8, α = 0.05).

| **Species** | **Temperature** | **statistics** | **df** | **adjusted p-values** |
| --- | --- | --- | --- | --- |
| ***S. pistillata*** | 28 | -2.518 | 7 | **0.04** |
|  | 30 | 0.191 | 7 | 0.854 |
|  | 32 | 0.085 | 7 | 0.935 |
|  | 34 | 1.125 | 7 | 0.298 |
| ***A.muricata*** | 28 | -1.021 | 7 | 0.341 |
|  | 30 | 3.917 | 7 | **0.006** |
|  | 32 | 3.147 | 7 | **0.016** |
|  | 34 | 2.138 | 7 | 0.07 |
| ***S. hystrix*** | 28 | 1.619 | 7 | 0.15 |
|  | 30 | 7.232 | 7 | **< 0.001** |
|  | 32 | 3.202 | 7 | **0.015** |
|  | 34 | -0.856 | 7 | 0.42 |
| ***M. dichotoma*** | 28 | 2.928 | 7 | **0.022** |
|  | 30 | -0.178 | 7 | 0.863 |
|  | 32 | -2.023 | 7 | 0.083 |
|  | 34 | 2.154 | 7 | 0.068 |
| ***P. verrucosa*** | 28 | 1.482 | 7 | 0.182 |
|  | 30 | -3.702 | 7 | **0.008** |
|  | 32 | -9.326 | 7 | **< 0.001** |
|  | 34 | -7.600 | 7 | **< 0.001** |
| ***P. lobata*** | 28 | 2.118 | 7 | 0.072 |
|  | 30 | 2.987 | 7 | **0.02** |
|  | 32 | 0.771 | 7 | 0.466 |
|  | 34 | -1.110 | 7 | 0.304 |

**Table S10. Component scores and most contributing physiological response variable from the Principal component analysis (n = 8, α = 0.05).**

|  | ***S. pistillata*** | ***A. muricata*** | ***S. hystrix*** | ***P. lobata*** | ***M. dichotoma*** | ***P. verrucosa*** |
| --- | --- | --- | --- | --- | --- | --- |
| **PC1** | *F_v_/F_m_*  (-0.57) | *F_v_/F_m_*  (-0.57) | *F_v_/F_m_*  (-0.55) | *rETR_max_*  (-0.61) | *rETR_max_*  (-0.59) | *rETR_max_*  (-0.57) |
| **PC2** | *R_dark_*  (0.87) | *NPQ/4_max_*  (0.77) | *R_dark_*  (-0.98) | *R_dark_*  (-0.64) | *Pg*  (0.65) | *R_dark_*  (-0.87) |

**Table S11. Summary statistics of the PERMANOVA performed for each coral species using the fixed factors ‘Treatment’ (ambient or elevated temperature) and ‘Location’ (GoA or GoT).** The test was conducted on Euclidian distances, using Bonferroni correction of the p-values. In bold, significant p-values (n = 8, α = 0.05).

| **Species** | **Source of variation** | **df** | **Sum of squares** | **Mean of squares** | **F model** | **R2** | **p-values** |
| --- | --- | --- | --- | --- | --- | --- | --- |
| *S. pistillata* | *Location* | 1 | 13.48 | 13.483 | 5.501 | 0.087 | **0.002** |
|  | *Treatment* | 1 | 60.081 | 60.081 | 24.516 | 0.388 | **< 0.001** |
|  | *Location:Treatment* | 1 | 12.816 | 12.816 | 5.229 | 0.083 | **0.004** |
|  | *Residuals* | 28 | 68.620 | 2.451 | NA | 0.443 | NA |
| *S. hystrix* | *Location* | 1 | 57.565 | 57.565 | 26.866 | 0.371 | **< 0.001** |
|  | *Treatment* | 1 | 29.897 | 29.897 | 13.952 | 0.193 | **< 0.001** |
|  | *Location:Treatment* | 1 | 7.542 | 7.542 | 3.520 | 0.049 | **0.028** |
|  | *Residuals* | 28 | 59.996 | 2.143 | NA | 0.387 | NA |
| *P. lobata* | *Location* | 1 | 36.668 | 36.668 | 10.997 | 0.237 | **< 0.001** |
|  | *Treatment* | 1 | 13.395 | 13.395 | 4.017 | 0.086 | **0.013** |
|  | *Location:Treatment* | 1 | 11.573 | 11.573 | 3.471 | 0.075 | **0.024** |
|  | *Residuals* | 28 | 93.364 | 3.334 | NA | 0.602 | NA |
| *A.muricata* | *Location* | 1 | 35.916 | 35.916 | 11.876 | 0.232 | **< 0.001** |
|  | *Treatment* | 1 | 31.869 | 31.869 | 10.538 | 0.206 | **< 0.001** |
|  | *Location:Treatment* | 1 | 2.538 | 2.538 | 0.839 | 0.016 | 0.511 |
|  | *Residuals* | 28 | 84.677 | 3.024 | NA | 0.546 | NA |
| *M. dichotoma* | *Location* | 1 | 69.513 | 69.513 | 60.373 | 0.448 | **< 0.001** |
|  | *Treatment* | 1 | 29.291 | 29.300 | 25.447 | 0.189 | **< 0.001** |
|  | *Location:Treatment* | 1 | 23.947 | 23.94 | 20.799 | 0.154 | **< 0.001** |
|  | *Residuals* | 28 | 32.239 | 1.151 | NA | 0.208 | NA |
| *P. verrucosa* | *Location* | 1 | 53.053 | 53.053 | 42.617 | 0.342 | **< 0.001** |
|  | *Treatment* | 1 | 36.644 | 36.644 | 29.436 | 0.236 | **< 0.001** |
|  | *Location:Treatment* | 1 | 30.446 | 30.446 | 24.457 | 0.196 | **< 0.001** |
|  | *Residuals* | 28 | 34.857 | 1.2449 | NA | 0.225 | NA |

**Table S12. Summary statistics of the pairwise permutations MANOVAs, performed for each coral species using the fixed factors ‘Treatment’ (ambient or elevated temperature) and ‘Location’ (GoA or GoT).** In bold, significant p-values (n = 8, α = 0.05).

| **Species** | **GoA Ambient : GoA Elevated temperature** | **GoT Ambient : GoT Elevated temperature** | **GoA Ambient : GoT Ambient** | **GoA Elevated temperature : GoT Elevated temperature** |
| --- | --- | --- | --- | --- |
| *S. pistillata* | **0.002** | **0.011** | **0.031** | **0.002** |
| *S. hystrix* | **0.0015** | **0.0015** | **0.003** | **0.0015** |
| *P. lobata* | **< 0.001** | **< 0.001** | **< 0.001** | **< 0.001** |
| *A. muricata* | **< 0.001** | **< 0.001** | **< 0.001** | **< 0.001** |
| *M. dichotoma* | **0.002** | **0.0012** | **0.0012** | **0.0012** |
| *P. verrucosa* | **< 0.001** | **< 0.001** | **< 0.001** | **< 0.001** |

**Table S13. Summary statistics of the one-sample Wilcoxon tests performed on the *T_opt_* of each species per location and their respective MMM, for *Pg* and *R_dark_*.** The p-values are indicated for each parameter. In bold, significant p-values (n = 8, α = 0.05).

|  |  | **GoA (MMM = 27.1°C)** | | **GoT (MMM = 30.9°C)** | |
| --- | --- | --- | --- | --- | --- |
|  | **Species** | **W** | **p-values** | **W** | **p-values** |
| Gross photosynthetic rate | *A. muricata* | 22 | 0.6406 | 20 | 0.8438 |
|  | *M. dichotoma* | 22 | 0.6406 | 1 | 1 |
|  | *P. verrucosa* | 34 | **0.0234** | 0 | **0.0078** |
|  | *P. lobata* | 36 | **0.0078** | 3 | 1 |
|  | *S. hystrix* | 3 | 0.5 | 0 | **0.0078** |
|  | *S. pistillata* | 36 | **0.0078** | 5 | 0.3125 |
| Dark respiration rate | *A. muricata* | 28 | **0.0156** | 35 | **0.0156** |
|  | *M. dichotoma* | 36 | **0.0138** | - | - |
|  | *P. verrucosa* | 36 | **0.01379** | - | - |
|  | *P. lobata* | 36 | **0.0078** | - | - |
|  | *S. hystrix* | 28 | **0.01073** | - | - |
|  | *S. pistillata* | 36 | **0.0078** | - | - |

**Table S14. Summary statistics of the Kruskal-Wallis tests performed on the *T_opt_* between all species per location, for *Pg* and *R_dark_*.** The p-values are indicated for each parameter. In bold, significant p-values (n = 8, α = 0.05).

|  | **Location** | **parameter** | **statistic** | **p-values** |
| --- | --- | --- | --- | --- |
| Between species  –  *T_opt_* based on *Pg* | *GoA* | 5 | 9.127 | 0.104 |
|  | *GoT* | 4 | 20.085 | **0.0005** |
| Between species  –  *T_opt_* based on *R_dark_* | *GoA* | 4 | 15.609 | **0.0036** |
|  | *GoT* | 2 | 6.214 | **0.0447** |

**Table S15. Summary statistics of the Kruskal-Wallis PostHOC test performed on the *T_opt_* between each species per location, for *Pg* and *R_dark_* when differences were found significant.** The p-values are indicated for each parameter. In bold, significant p-values (n = 8, α = 0.05).

| ***T_opt_ based on Pg - GoT*** | 1. *muricata* | *P. lobata* | *P. verrucosa* | *S. hystrix* |
| --- | --- | --- | --- | --- |
| *P. lobata* | 1.0 | - | - | - |
| *P. verrucosa* | **0.0014** | **0.006** | - | - |
| *S. hystrix* | 0.9688 | 0.9688 | 0.113 | - |
| *S. pistillata* | 1.0 | 1.0 | 0.2834 | 1.0 |

| ***T_opt_ based on R_dark_ - GoA*** | 1. *muricata* | *M. dichotoma* | *P. lobata* | *P. verrucosa* |
| --- | --- | --- | --- | --- |
| *M. dichotoma* | 0.965 | - | - | - |
| *P. lobata* | 0.965 | **0.048** | - | - |
| *P. verrucosa* | 0.965 | 1.0 | **0.048** | - |
| *S. pistillata* | 0.965 | **0.043** | 1.0 | **0.043** |

| ***T_opt_ based on R_dark_ - GoT*** | 1. *muricata* | *P. lobata* |
| --- | --- | --- |
| *P. lobata* | 0.071 | - |
| *S. hystrix* | 0.738 | 0.132 |


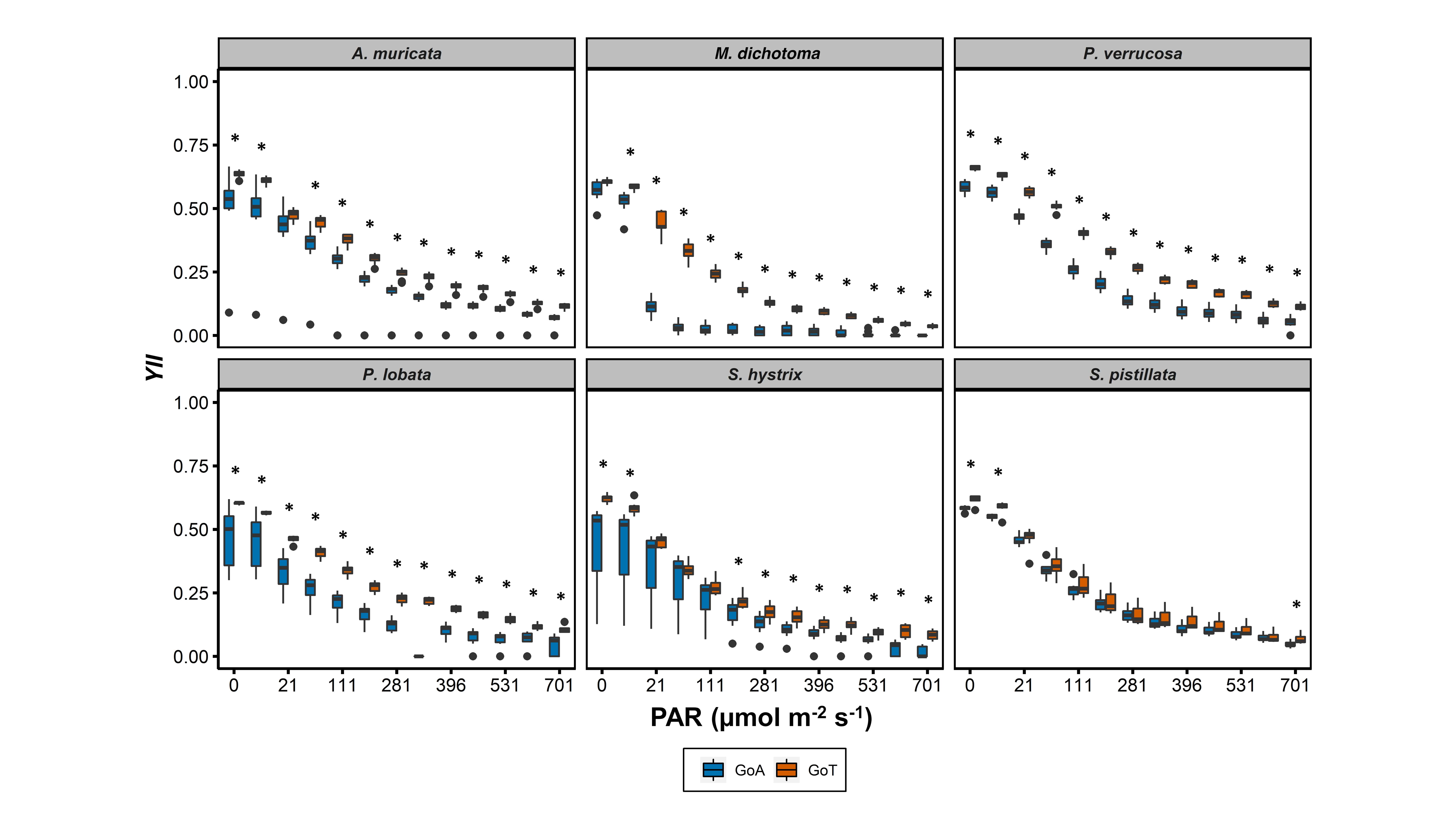


**Fig. S1 Effective quantum yield of PSII (YII) rapid light curve (RLC), for six reef-building coral species from two locations (Gulf of Aqaba (GoA) or Gulf of Tadjoura (GoT)) under ambient local temperatures (22°C and 28°C respectively).** PAR stands for photosynthetically active radiation. Star symbols (*) refer to significant differences between location at each PAR values based on Kruskal-Wallis rank sum tests (n = 8, α = 0.05). Black dots correspond to plots outliers. Error bars represent standard deviation.


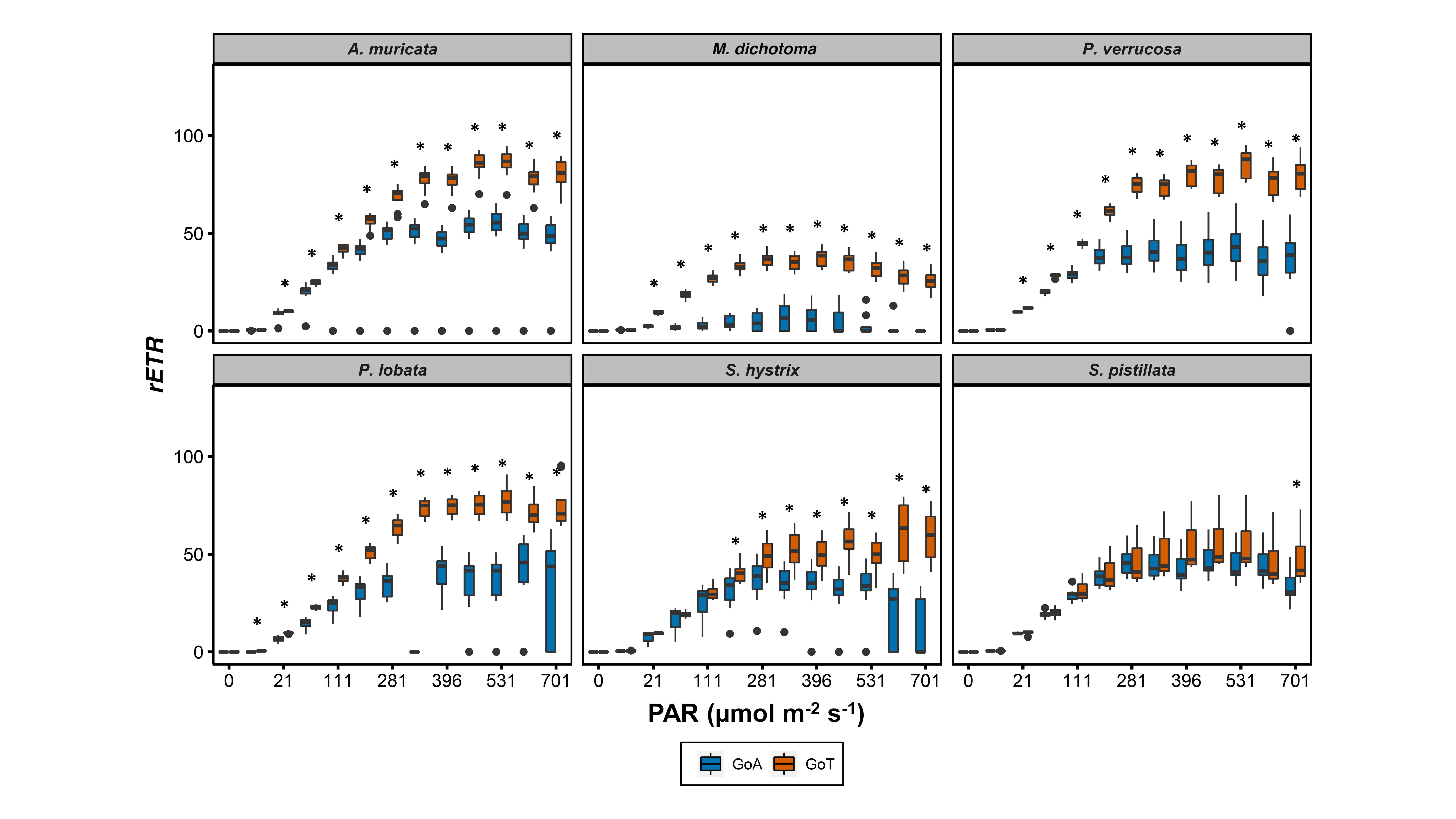


**Fig. S2 Relative electron transport rate (rETR) rapid light curve (RLC),** **for six reef-building coral species from two locations (Gulf of Aqaba (GoA) or Gulf of Tadjoura (GoT)) under ambient local temperatures (22°C and 28°C respectively).** PAR stands for photosynthetically active radiation. Star symbols (*) refer to significant differences between location at each PAR values based on Kruskal-Wallis rank sum tests (n = 8, α = 0.05). Black dots correspond to plots outliers. Error bars represent standard deviation.


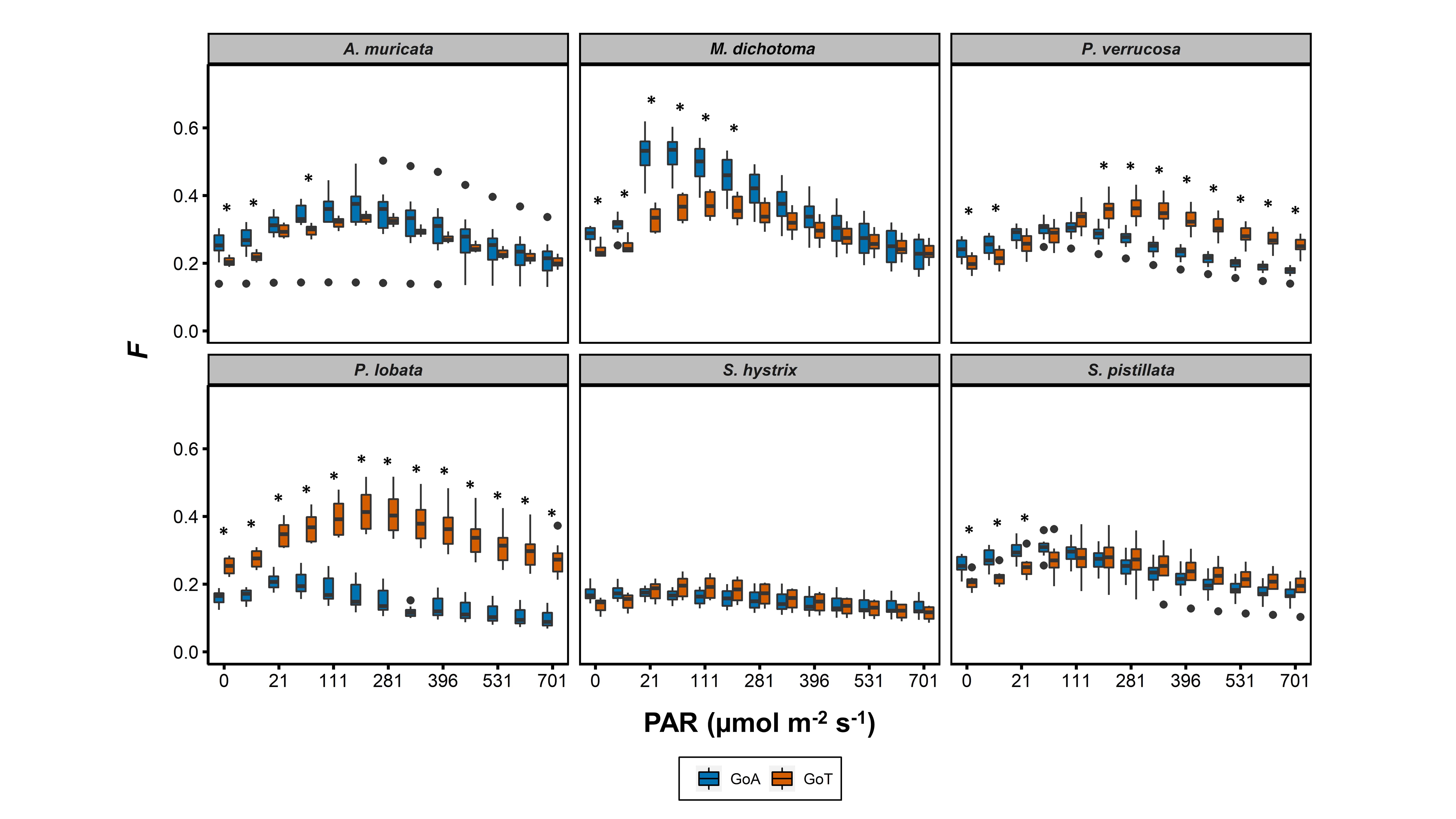


**Fig. S3** **Fluorescence (F) rapid light curve (RLC), for six reef-building coral species from two locations (Gulf of Aqaba (GoA) or Gulf of Tadjoura (GoT)) under ambient local temperatures (22°C and 28°C respectively). PAR stands for photosynthetically active radiation.** Star symbols (*) refer to significant differences between location at each PAR values based on Kruskal-Wallis rank sum tests (n = 8, α = 0.05). Black dots correspond to plots outliers. Error bars represent standard deviation.


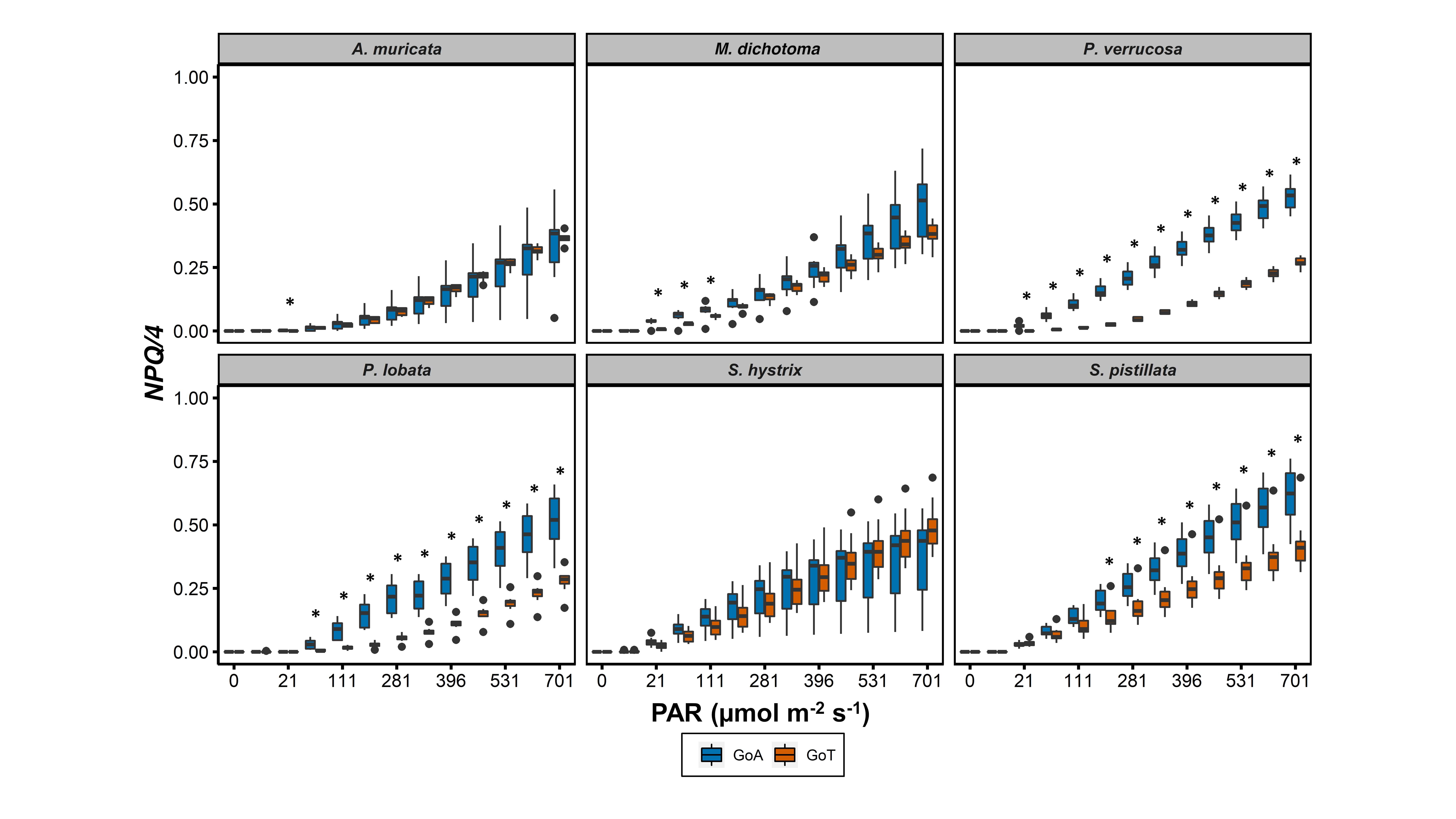


**Fig. S4** **Non-photochemical quenching (NPQ/4) rapid light curve (RLC), for six reef-building coral species from two locations (Gulf of Aqaba (GoA) or Gulf of Tadjoura (GoT)) under ambient local temperatures (22°C and 28°C respectively).** PAR stands for photosynthetically active radiation. Star symbols (*) refer to significant differences between location at each PAR values based on Kruskal-Wallis rank sum tests (n = 8, α = 0.05). Black dots correspond to plots outliers. Error bars represent standard deviation.


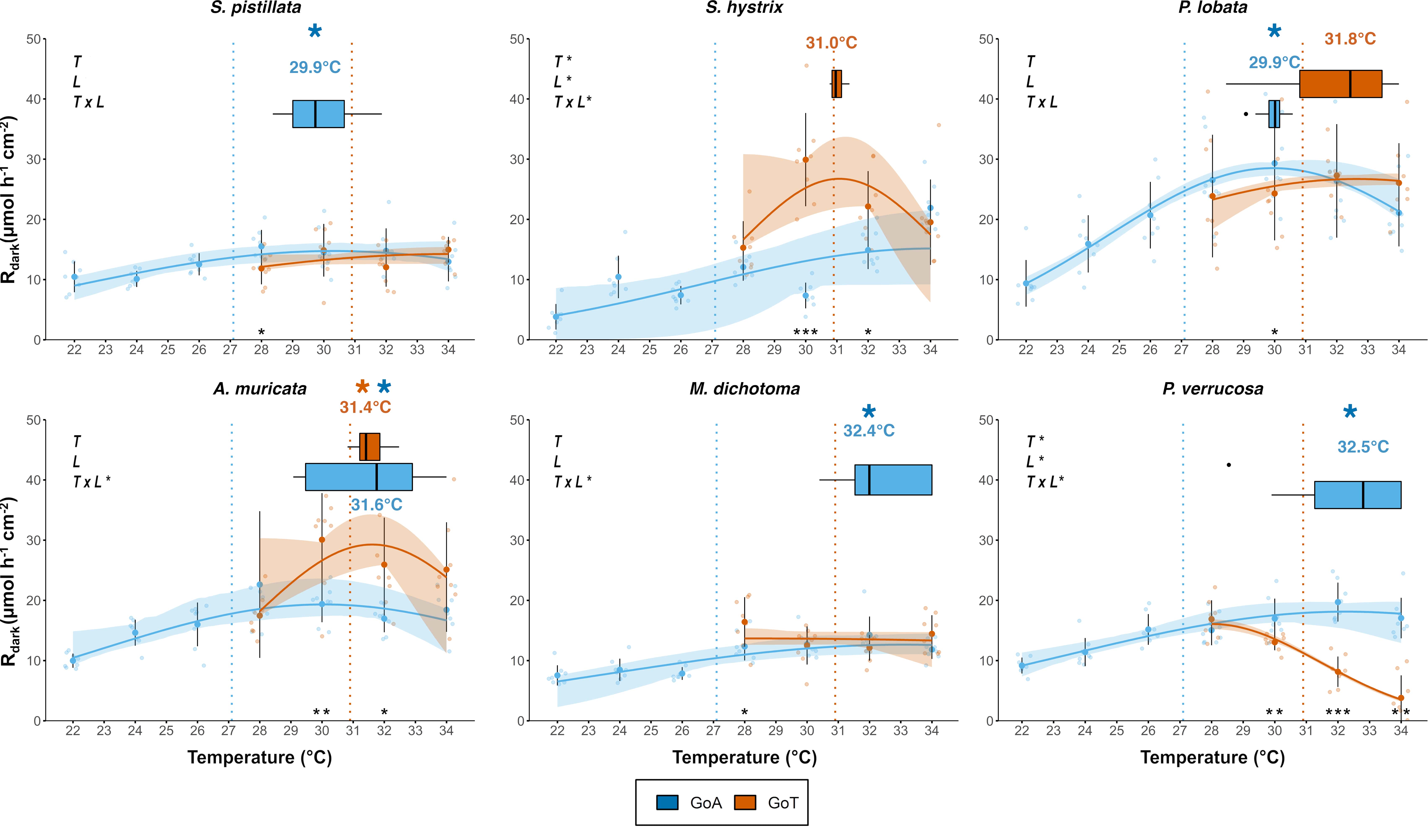


**Fig. S5 Dark respiration (*R_dark_*) thermal performance curves (TPC) and estimated thermal optima (*T_opt_*) of six scleractinian species from Gulf of Aqaba (GoA) or Gulf of Tadjoura (GoT), between local ambient temperatures (22°C and 28°C respectively) and 34°C (2°C above the summer maximum in the GoT).** Fit lines are predictions of *R_dark_* obtained from gaussian models, and confidence intervals are based on a non-parametric bootstrapping approach. Boxplots correspond to *T_opt_* derived from fitted models for each coral fragment. Temperatures above each box correspond to the average *T_opt_* (see also Table S5). Bold points represent means while transparent points correspond the raw *R_dark_* data. Dotted vertical lines indicate the monthly maximum mean seawater temperatures (MMM) in the GoA (27.1°C, blue) and in the GoT (30.9°C, orange). Results of repeated-measure ANOVAs are reported for each species using temperature (*T*) and location (*L*) as fixed factors and computing their interaction (*T x L*). Asterisks below the curves represent levels of significance of the post hoc pairwise t-test performed for each species between locations at each common temperature. Finally, asterisks to the left of the boxplots represent significant levels from Wilcoxon tests. *, **, *** respectively refer to significance levels of < 0.05, < 0.01 and < 0.001 (n = 8, α = 0.05). Black dots correspond to plots outliers. Error bars represent 95% confidence intervals.
